# Strophioglandins A–C, highly rearranged norditerpenoids with an unusual tricyclo[6.4.1.0^4,13^]tridecane core from *Strophioblachia glandulosa* var. *cordifolia*

**DOI:** 10.1007/s13659-025-00548-1

**Published:** 2025-09-15

**Authors:** Jian-Kai Xia, Lei-Ming Wu, Wei-Ye Wu, Dong Huang, Fang-Yu Yuan, Lei Li, Shu-Qi Wu, Yan-Jiang Zhang, Tao Yuan, Xin Chen, Gui-Hua Tang, Jia-Luo Huang, Sheng Yin

**Affiliations:** 1 School of Pharmaceutical Sciences, Sun Yat-Sen University, Guangzhou, 510006 China; 2 The Laboratory of Effective Substances of Jiangxi Genuine Medicinal Materials, College of Life Sciences, Jiangxi Normal University, Nanchang, 330022 China; 3 School of Life Science and Technology, Wuhan Polytechnic University, Wuhan, 430023 China

**Keywords:** *Strophioblachia glandulosa* var. *cordifolia*, Rearranged norditerpenoids, Anti-inflammatory effects, MAPK signaling pathways

## Abstract

Strophioglandins A−C (**1**−**3**), three highly rearranged norditerpenoids featuring an unusual tricyclo[6.4.1.0^4,13^]tridecane core, were isolated from *Strophioblachia glandulosa* var. *cordifolia*. Integrated spectroscopic analyses, X-ray crystallography, and ECD calculations synergistically determined their molecular architectures. Remarkably, all compounds manifested potent anti-inflammatory effects in LPS-activated RAW264.7 cells with IC_50_ values ranging from 7.83 ± 1.11 to 15.09 ± 1.21 μM. Mechanism study revealed that strophioglandin A (**1**), the most potent compound, could suppress the expression of multiple inflammatory factors by inhibiting the P38 and Erk1/2 MAPK signaling pathways.

**Graphical Abstract:**

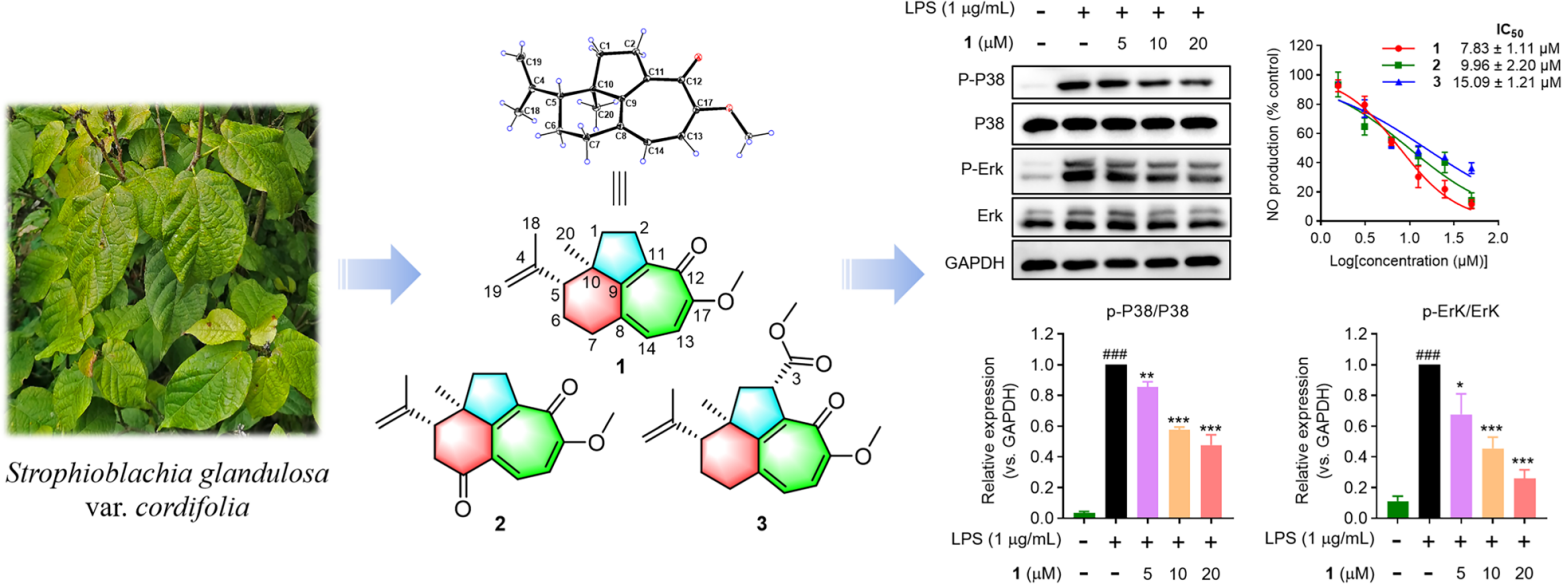

**Supplementary Information:**

The online version contains supplementary material available at 10.1007/s13659-025-00548-1.

## Introduction

Euphorbiaceae plants are well known for producing structurally intricate macrocyclic and polycyclic diterpenoids with a broad range of bioactivities [1–6]. The pharmacological and chemical significance of these diterpenoids drives sustained research interest, because they not only serve as potential leads in drug discovery but also pose formidable synthetic challenges that inspire novel methodology design in organic chemistry. For instance, ingenol mebutate, an ingenane ester isolated from *Euphorbia peplus* L., received Food and Drug Administration (FDA) approval in 2012 as a treatment for actinic keratosis [7]. Its precursor, ( +)-ingenol, featuring a 5/7/7/3 tetracyclic architecture, has been elaborately synthesized in only 14 steps using a two-phase strategy [8, 9]. (−)-Pepluanol B, a potassium channel inhibitor with an unusual 5/5/8/3 ring system, has been totally synthesized via an unprecedented bromo-epoxidation maneuver [10].

*Strophioblachia glandulosa* var. *cordifolia* is a shrub distributed in southern Yunnan, China. Its chemical constituents have not been investigated to date. Previous phytochemical investigations on other species within *Strophioblachia* (Euphorbiaceae) have identified a series of structurally unique diterpenoids, some of which exhibited cytotoxic, anti-inflammatory, proliferation inhibition, neuroprotective, and anti-myocardial hypertrophy activities [11–14].

**Fig. 1:**
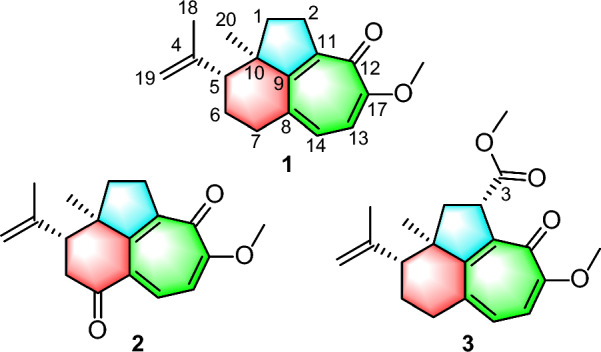
The structures of compounds **1**−**3**

As part of our ongoing quest for bioactive natural products [15–18], three highly rearranged norditerpenoids, strophioglandins A−C (**1**−**3**), featuring an unusual tricyclo[6.4.1.0^4,13^]tridecane core (Fig. 1), were isolated from the twigs and leaves of *S*. *glandulosa* var. *cordifolia*. Their structures, including the absolute configurations, were identified by HRESIMS, spectroscopic methods, X-ray crystallography, and ECD calculations. Herein, we detail their structural characterization, putative biosynthetic pathways, anti-inflammatory effects, and the underlying mechanism.

## Results and discussion

Strophioglandin A (**1**) was obtained as colorless needle crystals with a molecular formula C_18_H_22_O_2_ as determined by the HRESIMS ion at *m*/*z* 293.1518 [M + Na]^+^ (calcd. for C_18_H_22_O_2_Na^+^, 293.1512), indicating eight indices of hydrogen deficiency (IHDs). The ^1^H NMR data (Table 1) displayed signals for two methyl groups [(*δ*_H_ 1.02 (s) and 1.84 (s)], a methoxy [*δ*_H_ 3.89 (s)], two *cis*-olefinic protons [*δ*_H_ 6.65 (1H, d, *J* = 10.3 Hz) and 6.87 (1H, d, *J* = 10.3 Hz)], two terminal double bond protons [*δ*_H_ 4.77 (s) and 4.95 (s)], and a series of aliphatic multiplets. The 1D NMR data (Table 1) of **1** showed 18 carbon resonances ascribed to a ketone carbonyl (*δ*_C_ 177.1), four double bonds (*δ*_C_ 162.5, 157.1, 148.7, 146.3, 136.5, 130.8, 113.2, 112.2), a methoxy group (*δ*_C_ 56.1), two methyls, four *sp*^3^ methylenes, an *sp*^3^ methine, and a quaternary carbon. As five of the eight IHDs were consumed by four double bonds and a ketone carbonyl, the remaining three IHDs were indicative of a tricyclic ring system.

**Table 1:** ^1^H (400 MHz) and ^13^C (100 MHz) NMR data of **1**−**3** in CDCl_3_ (*δ* in ppm,* J* in Hz)

No	1		2		3	
	*δ*_H_, multi. (*J*)	*δ*_C_, type	*δ*_H_, multi. (*J*)	*δ*_C_, type	*δ*_H_, multi. (*J*)	*δ*_C_, type
1*α*	1.87, m	38.1, CH_2_	1.95, m	37.4, CH_2_	2.07, m	41.4, CH_2_
1*β*	1.89, m		1.98, m		2.37, m	
2	*α*, 2.95, overlapped *β*, 3.16, m	32.0, CH_2_	*α*, 3.06, m *β*, 3.24, m	32.8, CH_2_	4.32, d (11.0)	49.5, CH
3						174.7, C
4		146.3, C		143.6, C		145.7, C
5	2.29, dd (12.9, 3.9)	50.9, CH	2.83, m	49.3, CH	2.30, dd (12.6, 4.7)	51.1, CH
6*α*	2.06, m	24.6, CH_2_	2.91, m	39.4, CH	2.04, m	24.4, CH_2_
6*β*	1.80, m		2.62, dd (17.4, 3.0)		1.82, m	
7	*α*, 2.98, overlapped; *β*, 2.79, m	32.1, CH_2_		198.3, C	*α*, 3.01, m *β*, 2.79, m	31.4, CH_2_
8		136.5, C		129.2, C		136.1, C
9		157.1, C		157.3, C		157.8, C
10		52.2, C		52.2, C		52.6, C
11		148.7, C		147.7, C		144.3, C
12		177.1, C		176.9, C		176.8, C
13	6.65, d (10.3)	112.2, CH	6.86, d (10.7)	110.5, CH	6.64, d (10.3)	112.1, CH
14	6.87, d (10.3)	130.8, CH	8.09, d (10.7)	135.8, CH	6.93, d (10.3)	131.8, CH
17		162.5, C		166.9, C		163.1, C
18	1.84, s	24.4, CH_3_	1.88, s	24.2, CH_3_	1.84, s	23.9, CH_3_
19a	4.95, s	113.2, CH_2_	5.07, s	114.6, CH_2_	4.95, s	113.9, CH_2_
19b	4.77, s		4.86, s		4.80, s	
20	1.02, s	19.6, CH_3_	1.18, s	18.6, CH_3_	1.15, s	21.3, CH_3_
3-OMe					3.69, s	52.2, CH_3_
17-OMe	3.89, s	56.1, CH_3_	4.03, s	57.0, CH_3_	3.89, s	56.1, CH_3_

**Fig. 2:**
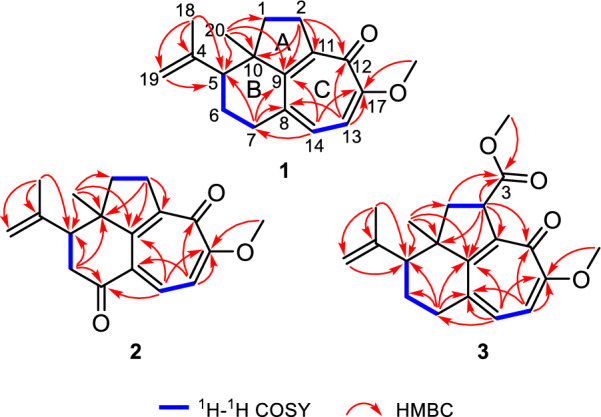
^1^H−^1^H COSY and key HMBC correlations of **1**−**3**

The planar structure of **1** was determined through comprehensive analysis of its 2D NMR data, including HSQC, ^1^H−^1^H COSY, and HMBC spectra. The key HMBC correlations from Me-20 to C-1/C-9/C-10 and from H_2_-2 to C-9/C-10/C-11, along with the ^1^H−^1^H COSY correlation of H_2_-1/H_2_-2 (Fig. 2), constructed a fragment of five-membered ring A with Me-20 located at C-10. Additionally,^1^H−^1^H COSY spectrum of H-5/H_2_-6/H_2_-7, together with the HMBC cross-peaks from Me-20 to C-5/C-9/C-10 and from H_2_-7 to C-5/C-8/C-9 instructively established a six-membered ring B, which was fused with ring A by sharing C-9 and C-10. A typical isopropenyl group was positioned at C-5 in ring B as elucidated by the HMBC correlations from Me-18 to C-5 and the terminal double bond (*δ*_C_ 113.2 and 146.3), as well as from H_2_-19 to C-5. The ^1^H−^1^H COSY correlation of H-13/H-14 and the HMBC correlations from H_2_-2 to C-9/C-11/C-12, from H-13 to C-8/C-12/C-17, from H-14 to C-7/C-9/C-17, and from OMe-17 to C-17, led to the confirmation of a tropolone nucleus (ring C) with a ketone carbonyl (*δ*_C_ 177.1) and a methoxy group (*δ*_C_ 56.1 and *δ*_H_ 3.89) assigned at C-12 and C-17, respectively. Hence, the planar structure of **1** was established (Fig. 2), featuring a rare tricyclo[6.4.1.0^4,13^]tridecane core.

**Fig. 3:**
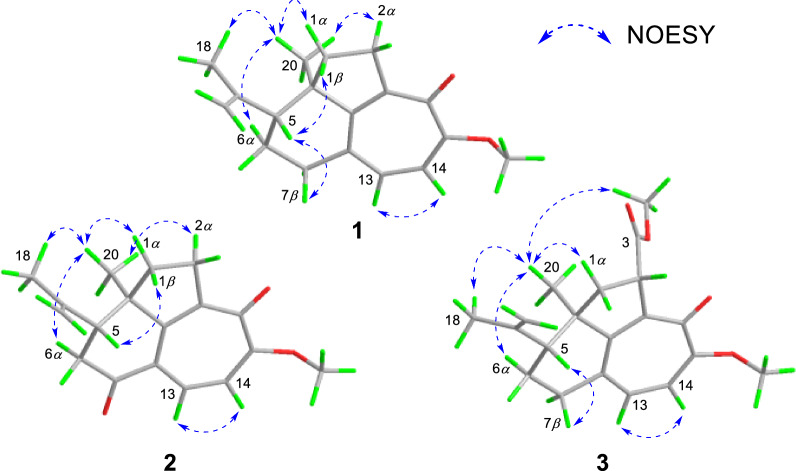
Key NOESY correlations of **1**−**3**

**Fig. 4:**
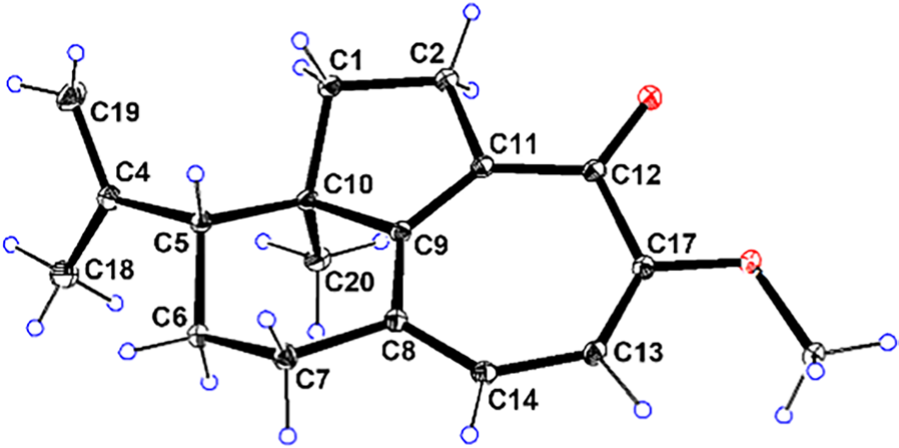
X-ray ORTEP diagram of **1**

NOESY experiment enabled determination of the relative configuration of compound **1** (Fig. 3). The NOESY spectrum revealed a cross-peak between Me-18 and Me-20, indicating spatial proximity between the isopropenyl group and Me-20. These groups were thus assigned as co-facial and *α*-oriented. Therefore, the relative stereochemistry 5R*,10R* was assigned to **1**. The 5*R*,10*R* absolute configuration of **1** was establishedby X-ray crystallography [Flack parameter: −0.06(10); CCDC number: 2422939] (Fig. 4).

**Fig. 5:**
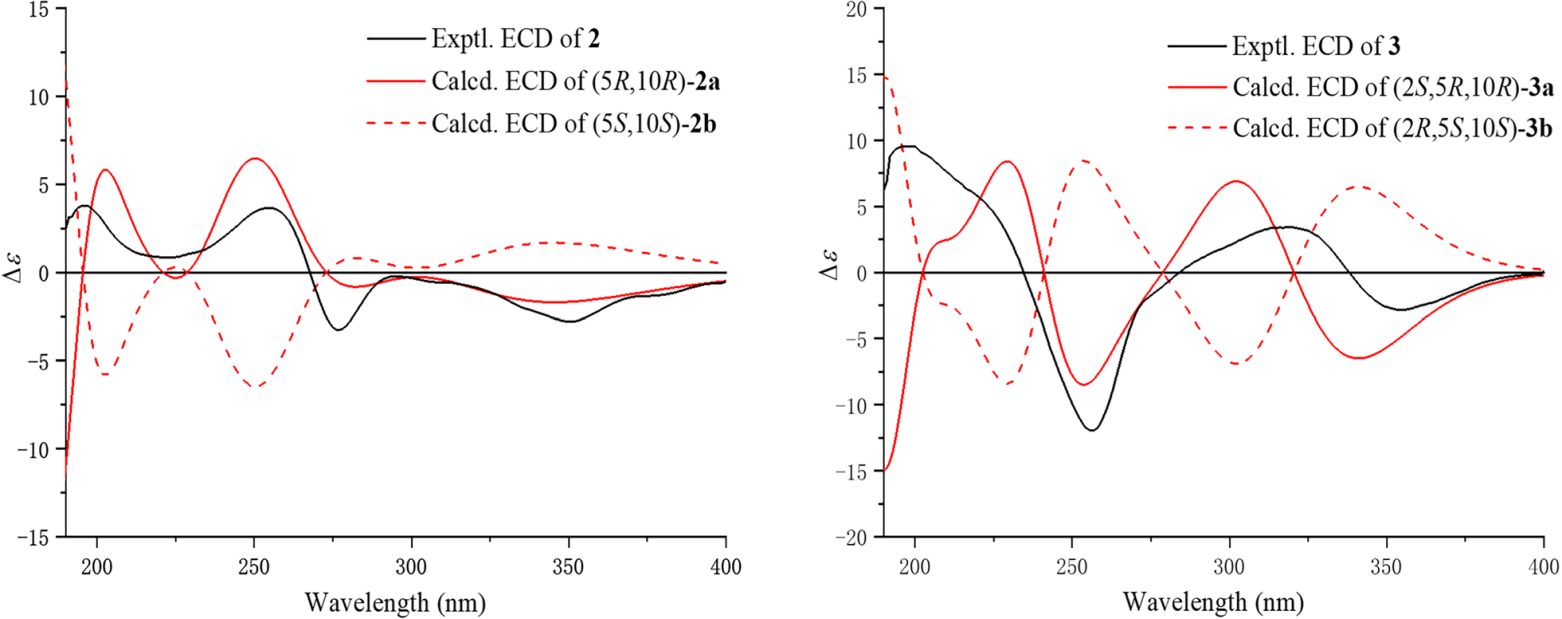
Experimental and calculated ECD curves of **2** and **3**

The HRESIMS data (*m*/*z* 307.1302 [M + Na]^+^, calcd. for C_18_H_20_O_3_Na^+^, 307.1305) established the molecular formula of strophioglandin B (**2**) as C_18_H_20_O_3_, implying nine IHDs. NMR data comparison (Table 1) indicated that **2** shares a core structure with **1**, differing only by oxidation of the C-7 methylene (CH₂-7) in **1** to the carbonyl group in **2**, which was identified by signals from H_2_-6 to C-5/C-7(*δ*_C_ 198.3)/C-10 and from H-14 to C-7 in its HMBC spectrum, as well as the cross-peak (H-5 and H_2_-6) in the ^1^H−^1^H COSY

experiment (Fig. 2). Spatial proximity between Me-20 and the isopropenyl moiety was confirmed by NOESY (Fig. 3), establishing their *α*-configurational assignment. Biosynthetic considerations suggest congruent absolute configuration between **1** and **2**, which was further supported by the comparable ECD curves of **2** and (5*R*,10*R*)-**2a** (Fig. 5).

The ion peak at *m*/*z* 329.1751 ([M + H]^+^, calcd. for C_20_H_25_O_4_^+^, 329.1747) in the HRESIMS spectrum assigned a molecular formula C_20_H_24_O_4_ to strophioglandin C (**3**). Comprehensive NMR data analysis established **3** as a structural analogue of **1**, differing only by the replacement of one hydrogen at CH₂-2 with a methoxycarbonyl group, which was substantiated by correlations from H-2 to C-3/C-9/C-10/C-11/C-12 and from H_3_-OMe-3 to C-3 (*δ*_C_ 174.7) in the HMBC experiment, coupled with the ^1^H−^1^H COSY cross-peak of H_2_-1/H-2 (Fig. 2). Therefore, we established the planar constitution of **3** as shown in Fig. 2. The NOESY spectrum showed key cross-peaks of Me-18/Me-20 and OMe-3/Me-20, implying they possessed spatial proximity and were arbitrarily defined as *α*-orientated. The relative stereochemistry 2S*,5R*,10R* was thus ascertained for **3**. The absolute configuration 2*S*,5*R*,10*R* was further given for** 3** as supported by the comparable ECD curves of **3** and (2*S*,5*R*,10*R*)-**3a** (Fig. 5).

**Scheme 1:**
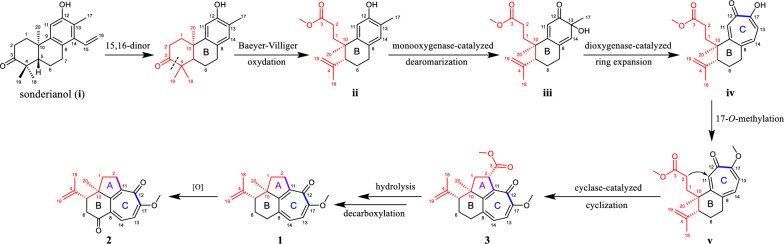
Proposed biosynthetic pathways for **1**−**3**

We propose putative biosynthetic pathways for **1**−**3** to get a better understanding of their structures (Scheme 1). First, sonderianol (**i**), a cleistanthane diterpenoid isolated from the same genus, was considered as a biosynthetic precursor, which may undergo carbon degradation and Baeyer–Villiger oxidation to form a 3,4-*seco* intermediate (**ii**). **ii** was then dearomatized via hydroxylation at C-13 catalyzed by monooxygenase [19]. A key tropolone nucleus (ring C) in **iv** was generated via dioxygenase-catalyzed ring expansion of **iii** [19]. The 17-*O*-methylation of **iv** afforded **v**, followed by the cyclase-catalyzed cyclization (attack from C-2 to C-11) to form compound **3** [20, 21]. Subsequently, **3** underwent hydrolysis and decarboxylation to afford **1**, which could further produce **2** by the oxidation at C-7.

**Fig. 6:**
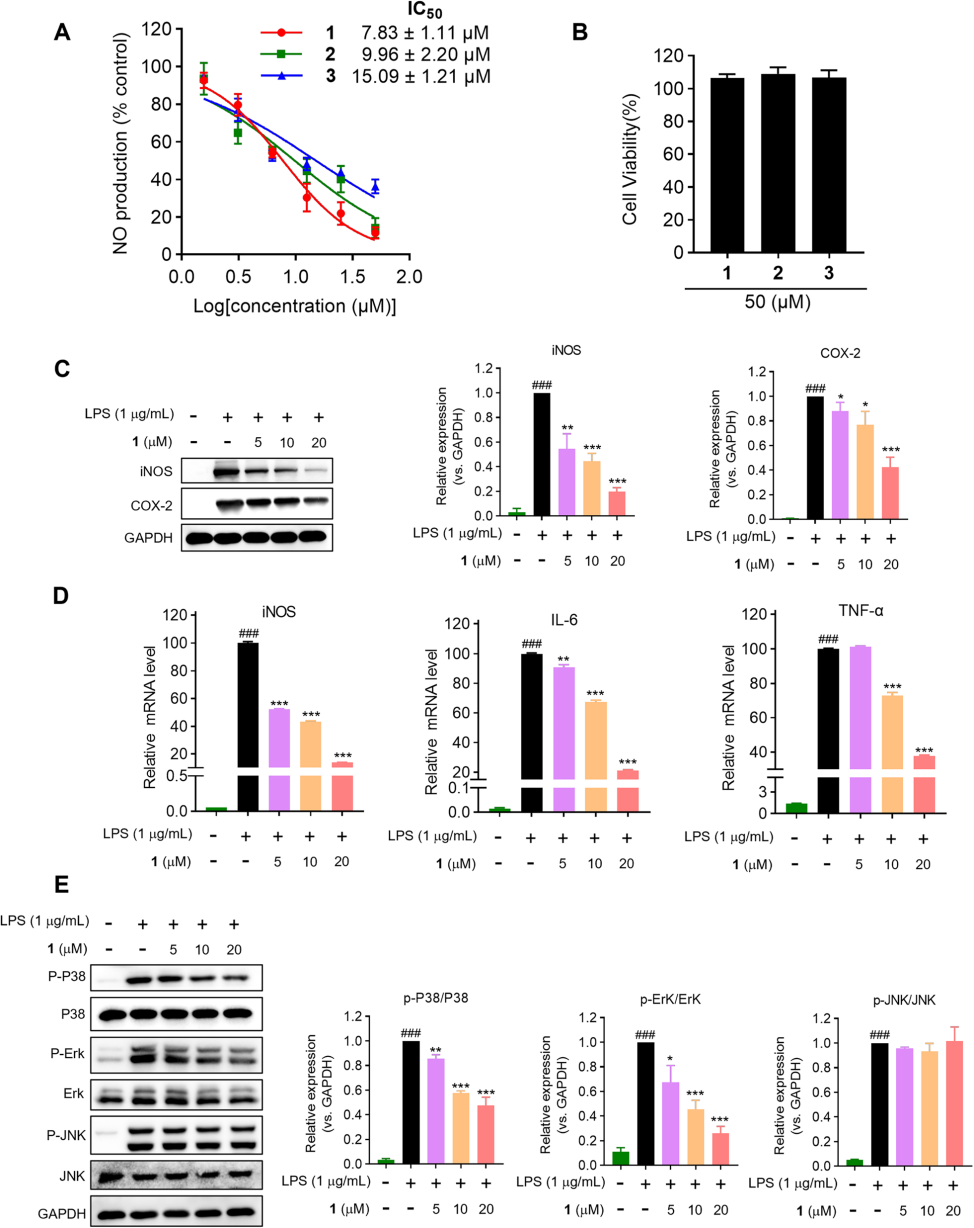
The anti-inflammatory effects of **1**−**3** in Raw264.7 cells. **A** Inhibitory effects of **1**−**3** on NO production. **B** The cytotoxicities of **1**−**3** measured by CCK-8 reagent. **C** Inhibitory effects of **1** on the expression of COX-2 and iNOS. **D** Inhibitory effects of **1** on mRNA levels by qRT-PCR analysis. **E** Immunoblot analysis of MAPK pathway proteins. The data were presented as the mean ± SD of at least three experiments. ^###^ *P* < 0.001 *vs* control group; ^*^ *P* < 0.05, ^**^ *P* < 0.01, ^***^ *P* < 0.001 *vs* LPS group

The suppression of lipopolysaccharide (LPS)-induced nitric oxide (NO) production in Raw264.7 macrophages by all isolates was measured to evaluate their anti-inflammatory effects. Preliminary screening suggested that **1**−**3** had potent inhibitory activities (IC_50_s = 7.83−15.09 μM) (Fig. 6A) compared with the recognized NO inhibitor, quercetin (IC_50_ = 14.55 μM) [22, 23]. Meanwhile, the non-cytotoxicities of **1**−**3** to Raw264.7 cells at 50 μM indicated that their inhibitory activities did not result from cytotoxic effect. (Fig. 6B). Pro-inflammatory mediators generated by macrophages are potential biomarkers in the process of inflammation, such as PGE2, NO, IL-6, and TNF-*α* [22]. Subsequently, the effects of the most potent compound, strophioglandin A (**1**), on mRNA and the proteins levels were investigated in Raw264.7 macrophages. Pretreatment of **1** could dose-responsively suppress the LPS-triggered iNOS/COX-2 overexpression (Fig. 6C). Additionally, dose-responsive suppression of iNOS/IL-6/TNF-*α* transcription by **1** at concentrations of 5, 10, and 20 μM was evidenced as shown in Fig. 6D. The experiments mentioned above demonstrated that **1** could suppress the up-regulation of multiple inflammation-associated factors in LPS-triggered murine macrophages.

Mitogen activated protein kinases (MAPK) signaling transduced by Toll-like receptors (TLRs) governs inflammatory effector expression in LPS-activated macrophages [24]. Consequently, Western blotting was employed to determine whether **1** could suppress the activated MAPK signaling pathways, specifically P38, Erk1/2, and JNK. As demonstrated in Fig. 6E, the pretreatment of **1** could dose-responsively constrain the P38 and Erk1/2 phosphorylation after brief (10 min) LPS-stimulation, while being ineffective against the JNK phosphorylation. These experiments verified that compound **1** could selectively curtail P38 and Erk1/2 phosphorylation (JNK unaffected), attenuating key inflammatory effectors expression.

## Experimental section

### General experimental procedures

These are provided in the Supporting Information.

### Plant material

We collected the twigs and leaves of *S. glandulosa* var. *cordifolia* from Yuanjiang County, Yunnan Province in August 2023. The plant was then identified by G.-H. Tang.

### Extraction and isolation

Twigs and leaves of *S. glandulosa* var. *cordifolia* (14.5 kg, dry weight) were powdered and extracted, under ambient conditions, thrice with 95% ethanol (3 × 75 L), generating a crude extract Weighing 1.2 kg. An aqueous suspension of the crude extract was subjected to liquid–liquid partition against petroleum ether (PE), followed by ethyl acetate (EtOAc), and then *n*-butanol (*n*-BuOH). Fractionation of the EtOAc extract (280.0 g) on D101 macroporous resin chromatographic column (CC), using MeOH/H₂O (3:1 → 1:0, *v*/*v*), yielded two fractions (Fr. I and Fr. II). Fr. I (86.2 g) was subjected to a silica gel column using CH₂Cl₂/MeOH (1:0 → 0:1, *v*/*v*), yielding eight subfractions (Fr. IA−Fr. IH). Among these, subfraction Fr. IB (10.0 g) was further subjected to silica gel chromatography using a PE/EtOAc gradient (8:1 → 0:1, *v*/*v*), affording nine fractions (Fr. IB_1_−Fr. IB_9_). Fr. IB_8_ (1.5 g) was further subjected to Sephadex LH-20 CC (MeOH), affording compounds** 1** (20.5 mg,* t*_R_ = 18.2 min), **2** (3.2 mg,* t*_R_ = 19.3 min), and **3** (16.8 mg,* t*_R_ = 15.6 min) followed by semi-preparative HPLC refinement (MeCN/H₂O, 48:52, *v*/*v*; 3.0 mL/min).

### Spectroscopic data of the compounds

#### Strophioglandin A (1)

Colorless crystal; ${[\alpha ]}_{D}^{20}$−160 (*c* 0.1, MeCN); UV (MeCN) *λ*_max_ (log *ε*) 253 (4.42), 333 (3.90) nm; ECD (*c* 7.40 × 10^−4^, MeCN) *λ*_max_ (Δ*ε*) 232 (+ 1.70), 354 (−3.07) nm; IR (neat)* ν*_max_ 2948, 2857, 1584, 1559, 1455, 1256, 1190, 1060, 1018, 890 cm^−1^; ^1^H and ^13^C NMR data, see Table 1; HRESIMS *m*/*z* 293.1518 [M + Na]^+^ (calcd. for C_18_H_22_O_2_Na^+^, 293.1512).

#### Strophioglandin B (2)

Yellowish oil; ${[\alpha ]}_{D}^{20}$−103 (*c* 0.1, MeCN); UV (MeCN) *λ*_max_ (log *ε*) 240 (4.16), 269 (4.31), 355 (3.89) nm; ECD (*c* 3.52 × 10^−4^, MeCN) *λ*_max_ (Δ*ε*) 196 (+ 3.80), 255 (+ 3.67), 277 (−3.26), 350 (−2.78) nm; IR (neat)* ν*_max_ 2960, 2925, 2853, 1683, 1585, 1456, 1261, 1187, 1055, 1007, 896 cm^−1^; ^1^H and ^13^C NMR data, see Table 1; HRESIMS *m*/*z* 307.1302 [M + Na]^+^ (calcd. for C_18_H_20_O_3_Na^+^, 307.1305).

#### Strophioglandin C (3)

Yellowish oil; ${[\alpha ]}_{D}^{20}$−55 (*c* 0.1, MeCN); UV (MeCN) *λ*_max_ (log *ε*) 251 (4.45), 330 (4.00) nm; ECD (*c* 3.05 × 10^−4^, MeCN) *λ*_max_ (Δ*ε*) 256 (−11.97), 319 (+ 3.43) nm; IR (neat)* ν*_max_ 2948, 1732, 1568, 1454, 1343, 1259, 1180, 1073, 1003, 894, 845 cm^−1^; ^1^H and ^13^C NMR data, see Table 1; HRESIMS *m*/*z* 329.1751 [M + H]^+^ (calcd. for C_20_H_25_O_4_^+^, 329.1747).

### Crystallographic data for 1

C_18_H_22_O_2_ (*M* = 270.35 g/mol): orthorhombic, space group *P* 2_1_ 2_1_ 2_1_ (no. 19),* a* = 6.19478(9) Å, *b* = 6.86349(9) Å, *c* = 34.2241(4) Å, *α* = 90°, *β* = 90°, *γ* = 90°, *V* = 1455.14(3) Å^3^, *Z* = 4, *T* = 100 K, *μ* (Cu Kα) = 0.616 mm^−1^, *D*_calc_ = 1.234 g/cm^−3^, 14 428 reflections measured (5.164° ≤ 2*θ* ≤ 157.466°), 3052 unique (*R*_int_ = 0.0449, *R*_sigma_ = 0.0436), which were used in all calculations. The final *R*_1_ was 0.0436 (*I* > 2*σ*(*I*)) and *wR*_2_ was 0.1176 (all data). Flack parameter:−0.06(10). CCDC number: 2422939.

### ECD calculations

These are provided in the Supporting Information for **2** and **3**.

### Cell culture

Cell culture was performed according to the established protocol [25].

### Cytotoxicity assay

Raw264.7 cells (5 × 10^4^ cells/well), prior to compounds treatment for 24 h, were allowed to adhere in 96-well plates. Following 24 h incubation at 37 °C, cell viability was assessed by using CCK-8 reagent (Dojindo, Japan) with 10 *μ*L reagent added per well. Absorbance was finally recorded by means of a multifunction microplate reader at 450 nm.

### Analysis of NO production

Following a 24-h incubation period after plating Raw264.7 macrophages (5 × 10^4^ cells/well) in 96-well plates, compounds were added at increasing concentrations (5, 10, and 20 μM) and incubated, with or without LPS (1 μg/mL), for 24 h in media. NO concentration in the culture medium was quantified using a Griess reagent kit, following the manufacturer’s protocol. For the assay, 50 *μ*L of Griess reagent was mixed with an equal volume of cell culture supernatant. With quercetin serving as the positive control, the absorbance at 540 nm, by means of a multifunction microplate reader, was measured.

### qRT-PCR analysis

This was performed according to the established protocol [25].

### Western blotting analysis

This was performed according to the established protocol [25].

### Statistical analysis

Statistical analysis followed the approach of reference [25].

## Conclusion

In summary, three highly rearranged cleistanthane norditerpenoids (**1**−**3**) featuring an unusual tricyclo[6.4.1.0^4,13^]tridecane carbon core, were isolated from *S. glandulosa* var. *cordifolia*. Compounds **1** and **2** are the first reported trinorditerpenoids with a highly fused 5/7/6-tricyclic skeleton. Notably, bioactivity evaluation demonstrated that all isolates exhibited remarkable anti-inflammatory effects. Strophioglandin A (**1**), the most potent compound, attenuated LPS-induced secretion of key pro-inflammatory cytokines (iNOS, IL-6, and TNF-*α*) in murine macrophages via blockade of MAPK signaling (P38 and Erk1/2 phosphorylation). This study deepens the chemical investigation of the genus *Strophioblachia*, and suggests potential application of the norditerpenoids as novel anti-inflammatory agents for inflammatory disease treatment.

## Supplementary Information

Additional file 1. The 1D and 2D NMR, HRESIMS, and IR spectra of 1 − 3, and ECD calculations data for 2 and 3.

## Data Availability

The experimental data supporting this work are accessible within the article and its Additional file 1.
