# Supplementary material for: Strophioglandins A–C, highly rearranged norditerpenoids with an unusual tricyclo[6.4.1.04,13]tridecane core from Strophioblachia glandulosa var. cordifolia
Source: Nat Prod Bioprospect. 2025 Sep 15;15(1):65. doi: 10.1007/s13659-025-00548-1 (PMC12436247; doi:10.1007/s13659-025-00548-1)

Supporting Information

Strophioglandins A−C, highly rearranged norditerpenoids with an unusual tricyclo[6.4.1.0^{4,13}]tridecane core from *Strophioblachia glandulosa* var. *cordifolia*

Jian-Kai Xia,† Lei-Ming Wu,† Wei-Ye Wu,† Dong Huang,† Fang-Yu Yuan,† Lei Li,† Shu-Qi Wu,† Yan-Jiang Zhang,† Tao Yuan,‡ Xin Chen,§ Gui-Hua Tang,† Jia-Luo Huang,*,† and Sheng Yin*,†

†School of Pharmaceutical Sciences, Sun Yat-sen University, Guangzhou 510006, People's Republic of China

‡The Laboratory of Effective Substances of Jiangxi Genuine Medicinal Materials, College of Life Sciences, Jiangxi Normal University, Nanchang 330022, People's Republic of China

§School of Life Science and Technology, Wuhan Polytechnic University, Wuhan 430023, People's Republic of China

*Corresponding authors. Tel/Fax: +86-20-39943090.

E-mail addresses: huangjluo3@mail.sysu.edu.cn (J.-L. Huang), yinsh2@mail.sysu.edu.cn (S. Yin).

Contents

1. Experimental Section 3
1.1 General experimental procedures 3
1.2 ECD calculations for assigning the absolute configurations of **2** and **3** 3
1.3 Conformers and energy analysis 4
1.4 Calculated ECD data 5
1.5 Crystal data and structure refinement for **1** 7
2. 1D and 2D NMR, HRESIMS, and IR spectra of 1−3 8
Figure S4. ^{1}H NMR spectrum of **1** in $CDCl_3$ (400 MHz) 8
Figure S5. ^{13}C NMR and DEPT 135° spectra of **1** in $CDCl_3$ (100 MHz) 8
Figure S6. $^{1}H-^{1}H$ COSY spectrum of **1** in $CDCl_3$ (400 MHz) 9
Figure S7. HSQC spectrum of **1** in $CDCl_3$ (400 MHz) 9
Figure S8. HMBC spectrum of **1** in $CDCl_3$ (400 MHz) 10
Figure S9. NOESY spectrum of **1** in $CDCl_3$ (400 MHz) 10
Figure S10. HRESIMS spectrum of **1** 11
Figure S11. IR spectrum of **1** 11
Figure S12. ^{1}H NMR spectrum of **2** in $CDCl_3$ (400 MHz) 12
Figure S13. ^{13}C NMR and DEPT 135° spectra of **2** in $CDCl_3$ (100 MHz) 12
Figure S14. $^{1}H-^{1}H$ COSY spectrum of **2** in $CDCl_3$ (400 MHz) 13
Figure S15. HSQC spectrum of **2** in $CDCl_3$ (400 MHz) 13
Figure S16. HMBC spectrum of **2** in $CDCl_3$ (400 MHz) 14
Figure S17. NOESY spectrum of **2** in $CDCl_3$ (400 MHz) 14
Figure S18. HRESIMS spectrum of **2** 15
Figure S19. IR spectrum of **2** 15
Figure S20. ^{1}H NMR spectrum of **3** in $CDCl_3$ (400 MHz) 16
Figure S21. ^{13}C NMR and DEPT 135° spectra of **3** in $CDCl_3$ (100 MHz) 16
Figure S22. $^{1}H-^{1}H$ COSY spectrum of **3** in $CDCl_3$ (400 MHz) 17
Figure S23. HSQC spectrum of **3** in $CDCl_3$ (400 MHz) 17
Figure S24. HMBC spectrum of **3** in $CDCl_3$ (400 MHz) 18
Figure S25. NOESY spectrum of **3** in $CDCl_3$ (400 MHz) 18
Figure S26. HRESIMS spectrum of **3** 19
Figure S27. IR spectrum of **3** 19

1. Experimental Section

1.1 General experimental procedures

The optical rotations were recorded by an MCP 200 modular circular polarimeter (Anton Paar). The ECD and UV data were tested by an Applied Photophysics Chirascan spectrometer. The IR spectra were determined on a Bruker Tensor 37 infrared spectrophotometer. The NMR spectra were measured on a Bruker AM-400 spectrometer at 25°C. HRESIMS were carried out on a Waters/SCIEX micromass Q-TOF spectrometer. X-ray data were obtained using an Agilent Xcalibur Nova X-ray diffractometer. Semipreparative HPLC was performed with a Shimadzu LC-20 AT equipped with an SPD-M20A PDA detector. A YMC-pack ODS-A column (250 × 10 mm, S-5 μm, 12 nm) was used for HPLC purification. Silica gel (100−200 and 300−400 mesh, Qingdao Haiyang Chemical Co., Ltd.), D101 macroporous adsorptive resins (Sinopharm Chemical Reagent Co., Ltd.), and Sephadex LH-20 gel (Amersham Biosciences) were used for column chromatography (CC). All solvents were of analytical grade (Guangzhou Chemical Reagents Co., Ltd.).

1.2 ECD calculations for assigning the absolute configurations of 2 and 3

The absolute configurations of **2** and **3** were determined by quantum chemical calculations of their theoretical ECD spectra. One of the two enantiomers for each compound, (5*R*,10*R*)-**2a** and (2*S*,5*R*,10*R*)-**3a** (Figure S1), were arbitrary chosen for theoretical studies. Conformational analyses were first carried out via Monte Carlo searching using molecular mechanism with MMFF force field in the Spartan 18 program.[1] The results showed six lowest energy conformers for **2a** and one for **3a** within an energy window of 2.5 kcal/mol. These conformers were reoptimized using DFT at the B3LYP/6-31G(d) level in gas phase using the Gaussian 09 program.[2] Four conformers of **2a** (Figure S2) and one of **3a** (Figure S3) with relative Gibbs free energies in the range of 0−1.5 kcal/mol were refined and considered for the next step. All the reoptimized conformers mentioned above for **2a** and **3a** were applied for theoretical ECD calculation. The energies, oscillator strengths, and rotational strengths of the first 30 electronic excitations were calculated using the TD-DFT methodology at the M062X/TZVP level in PCM (acetonitrile). The ECD spectra were simulated by the overlapping Gaussian function (σ = 0.40 eV, +15 nm in horizontal axis for **2a** and σ = 0.35 eV, +15 nm in horizontal axis for **3a**.[3] To get the final ECD spectrum of each compound, the simulated spectra of the lowest energy conformers were averaged according to the Boltzmann distribution theory and their relative Gibbs free energy (ΔG). The theoretical ECD curves of (5*S*,10*S*)-**2b** and (2*R*,5*S*,10*S*)-**3b** were obtained by directly reversing those of (5*R*,10*R*)-**2a** and (2*S*,5*R*,10*R*)-**3a**, respectively.

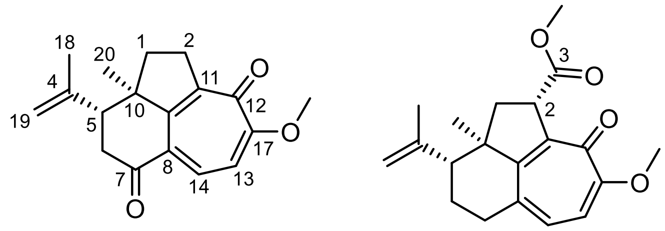

2a **3a**

Figure S1. Structures of **2a** and **3a**

References

(1) *Spartan 18*; Wavefunction Inc.:Irvine, CA.

(2) *Gaussian 09*, Revision A.1, Frisch, M. J.; Trucks, G. W.; Schlegel, H. B.; Scuseria, G. E.; Robb, M. A.; Cheeseman, J. R.; Scalmani, G.; Barone, V.; Mennucci, B.; Petersson, G. A.; Nakatsuji, H.; Caricato, M.; Li, X.; Hratchian, H. P.; Izmaylov, A. F.; Bloino, J.; Zheng, G.; Sonnenberg, J. L.; Hada, M.; Ehara, M.; Toyota, K.; Fukuda, R.; Hasegawa, J.; Ishida, M.; Nakajima, T.; Honda, Y.; Kitao, O.; Nakai, H.; Vreven, T.; Montgomery, Jr., J. A.; Peralta, J. E.; Ogliaro, F.; Bearpark, M.; Heyd, J. J.; Brothers, E.; Kudin, K. N.; Staroverov, V. N.; Kobayashi, R.; Normand, J.; Raghavachari, K.; Rendell, A.; Burant, J. C.; Iyengar, S. S.; Tomasi, J.; Cossi, M.; Rega, N.; Millam, J. M.; Klene, M.; Knox, J. E.; Cross, J. B.; Bakken, V.; Adamo, C.; Jaramillo, J.; Gomperts, R.; Stratmann, R. E.; Yazyev, O.; Austin, A. J.; Cammi, R.; Pomelli, C.; Ochterski, J. W.; Martin, R. L.; Morokuma, K.; Zakrzewski, V. G.; Voth, G. A.; Salvador, P.; Dannenberg, J. J.; Dapprich, S.; Daniels, A. D.; Farkas, Ö.; Foresman, J. B.; Ortiz, J. V.; Cioslowski, J.; Fox, D. J. Gaussian, Inc., Wallingford CT, 2009.

(3) Grimblat, N.; Zanardi, M. M.; Sarotti, A. M. *J. Org. Chem.* **2015**, *80*, 12526–12534.

1.3 Conformers and energy analysis

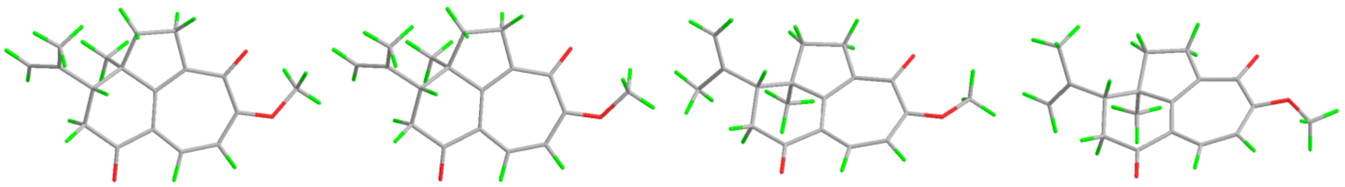

C1 C2 C3 C4

Figure S2. B3LYP/6-31G(d) optimized lowest energy conformers for **2a**.

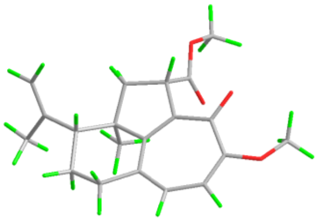

C1

Figure S3. B3LYP/6-31G(d) optimized lowest energy conformers for **3a**.

Table S1. **Energy (298.15 K) analysis for 2a and 3a**

Conf.	G (Hartree)	ΔG (kcal/mol)	Boltzmann Distribution
**2a**			
C1	-923.284488	0	1
C2	-923.284238	0.1568775	0.767271946
C3	-923.283565	0.57919173	0.376038998
C4	-923.283447	0.65323791	0.331840177
**3a**			
C1	-1077.078069	0	1

1.4 Calculated ECD data

ECD spectrum of each conformation is simulated according to the overlapping Gaussian functions expressed as:

$$\Delta\varepsilon(E)=\frac{1}{2.296\times10^{-39}\sqrt{\pi}\sigma}\sum_{i}^{A}\Delta E_i R_i e^{[-(E-\Delta Ei)^2/\sigma^2]}$$

Where σ is half the bandwidth at 1/e peak height and expressed in energy units. The parameters ΔE_i and R_i are the excitation energies and rotational strengths for the transition *i*, respectively.

The above function is converted to Δε, λ (wavelength) correlations as:

$$\Delta\varepsilon(\lambda)=\frac{1}{2.296\times10^{-39}\sqrt{\pi}\sigma}\sum_{i}^{A}\Delta E_i R_i e^{[-(1240/\lambda-\Delta Ei)^2/\sigma^2]}$$

and then simulation was accomplished by using the Excel 2003 and the Origin 9.1 software.

To get the final spectra, all the simulated spectra of conformations of each compound were averaged according to their energy and the Boltzmann distribution theory expressed as: $\frac{N_i^*}{N}=\frac{g_i e^{-\varepsilon_i/k_BT}}{\sum g_i e^{-\varepsilon_i/k_BT}}$

Table S2. Calculated ECD data for 2a in PCM

state	C1		C2		C3		C4	
	Excitation energies(eV)	Rotatory Strengths*	Excitation energies(eV)	Rotatory Strengths*	Excitation energies(eV)	Rotatory Strengths*	Excitation energies(eV)	Rotatory Strengths*
1	3.4818	-2.2241	3.4958	-11.1938	3.4805	-8.4338	3.6486	25.8956

2	3.63	1.5216	3.6287	-0.5168	3.6386	-0.2357	3.6575	-31.0953
3	3.8866	-19.4989	3.8884	-15.2364	3.8814	-19.1405	4.007	-15.0485
4	3.9725	-12.2983	3.9908	43.7222	3.9751	-18.2679	4.1322	12.5905
5	4.7061	19.0638	4.7172	22.3165	4.6887	16.4448	4.8537	9.6982
6	4.7955	-27.2482	4.8151	-82.0909	4.8212	19.7657	4.9601	-51.1722
7	5.1513	55.8818	5.1616	47.4656	5.089	4.8924	5.2657	60.3326
8	5.4869	30.1202	5.5174	7.1525	5.4943	4.5963	5.5751	14.8234
9	5.633	-12.329	5.6539	-8.5214	5.684	-30.2011	5.6505	-11.7487
10	5.9472	-0.7606	5.9474	-3.3359	5.8863	18.7556	6.0123	-12.2069
11	5.9595	-12.5813	6.0223	-15.4593	5.9487	5.3329	6.088	5.8094
12	6.1123	10.1874	6.1083	8.0099	6.0585	9.761	6.1608	-2.4816
13	6.285	7.4633	6.2788	0.8621	6.282	8.4055	6.3691	1.2078
14	6.428	-0.17	6.3688	-8.5134	6.4161	1.798	6.5307	-1.7339
15	6.5388	-7.5251	6.585	4.9573	6.5255	-6.7333	6.6646	-2.809
16	6.6535	19.5349	6.6531	25.2039	6.6752	12.762	6.7225	11.3155
17	6.7267	-2.2851	6.7275	-3.6623	6.764	4.1939	6.7532	5.2011
18	6.8093	27.9793	6.8306	16.3561	6.8178	-0.5416	6.8335	27.9439
19	6.9126	5.8316	6.9249	21.9157	6.8906	15.2478	6.9274	5.461
20	7.0379	-6.0938	7.0291	34.8293	7.0084	18.5744	7.0672	-23.6241
21	7.0462	-26.2127	7.0487	-27.3374	7.0355	-7.8173	7.1145	19.4399
22	7.153	30.8044	7.1539	-12.6929	7.141	-53.2144	7.1838	0.6537
23	7.1845	-18.8635	7.1972	-81.1138	7.1505	-3.0051	7.2368	-126.4129
24	7.2005	-58.6105	7.2448	11.6809	7.1675	25.478	7.2597	33.8892
25	7.2345	0.8602	7.2484	1.8171	7.2571	-0.1604	7.2966	-4.3407
26	7.2798	0.8632	7.3015	4.4426	7.3073	8.0659	7.3275	26.6528
27	7.3485	33.2239	7.3533	35.1367	7.3772	73.2869	7.4343	18.583
28	7.3637	-85.7718	7.377	-76.0672	7.4058	-38.435	7.4732	-53.0135
29	7.4865	11.7213	7.4675	1.0694	7.4786	7.0535	7.5141	8.9721
30	7.5608	2.8075	7.499	-19.4648	7.5468	-18.5847	7.5731	64.6156

* R(velocity)   10**-40 erg-esu-cm

Table S3. Calculated ECD data for 3a in PCM

C1					
state	Excitation energies(eV)	Rotatory Strengths*	state	Excitation energies(eV)	Excitation energies(eV)

1	3.8064	-13.9435	16	6.8762	-13.7176
2	4.0266	-87.0086	17	6.9022	-67.3431
3	4.1562	98.2903	18	6.9129	-1.1825
4	4.9001	-3.6982	19	6.9414	58.2706
5	5.2638	-33.6193	20	7.024	26.4419
6	5.4405	-14.8552	21	7.086	-61.0047
7	5.6206	29.3797	22	7.1527	-2.2751
8	5.6972	13.586	23	7.1764	5.2669
9	5.9729	-0.4603	24	7.2347	4.0378
10	6.1571	18.321	25	7.3004	-10.8615
11	6.307	-14.5779	26	7.375	-11.4628
12	6.5172	-9.2589	27	7.4444	13.5571
13	6.5849	-8.3433	28	7.4932	4.28
14	6.603	42.3195	29	7.5164	-1.944
15	6.8039	0.3009	30	7.5577	-0.7038

* R(velocity)   10**-40 erg-esu-cm

1.5 Crystal data and structure refinement for 1

Empirical formula	$C_{18}H_{22}O_2$
Formula weight	270.35
Temperature/K	100.00(10)
Crystal system	orthorhombic
Space group	$P2_12_12_1$
a/Å	6.19478(9)
b/Å	6.86349(9)
c/Å	34.2241(4)
α/°	90
β/°	90
γ/°	90
Volume/Å^3	1455.14(3)
Z	4
ρ_{calc}mg/mm^3	1.234
μ/mm^{-1}	0.616
F(000)	584.0
Crystal size/mm^3	0.15 × 0.05 × 0.05
2Θ range for data collection	5.164 to 157.466°
Index ranges	-7 ≤ h ≤ 7, -7 ≤ k ≤ 8, -42 ≤ l ≤ 43
Reflections collected	14428
Independent reflections	3052[R(int) = 0.0477]
Data/restraints/parameters	3052/0/192
Goodness-of-fit on F^2	1.060
Final R indexes [I>=2σ (I)]	R_1 = 0.0436, wR_2 = 0.1164
Final R indexes [all data]	R_1 = 0.0449, wR_2 = 0.1176
Largest diff. peak/hole / e Å^{-3}	0.31/-0.20
Flack parameter	-0.06(10)

2. 1D and 2D NMR, HRESIMS, and IR spectra of 1−3

Figure S4. ^{1}H NMR spectrum of **1** in $CDCl_3$ (400 MHz)

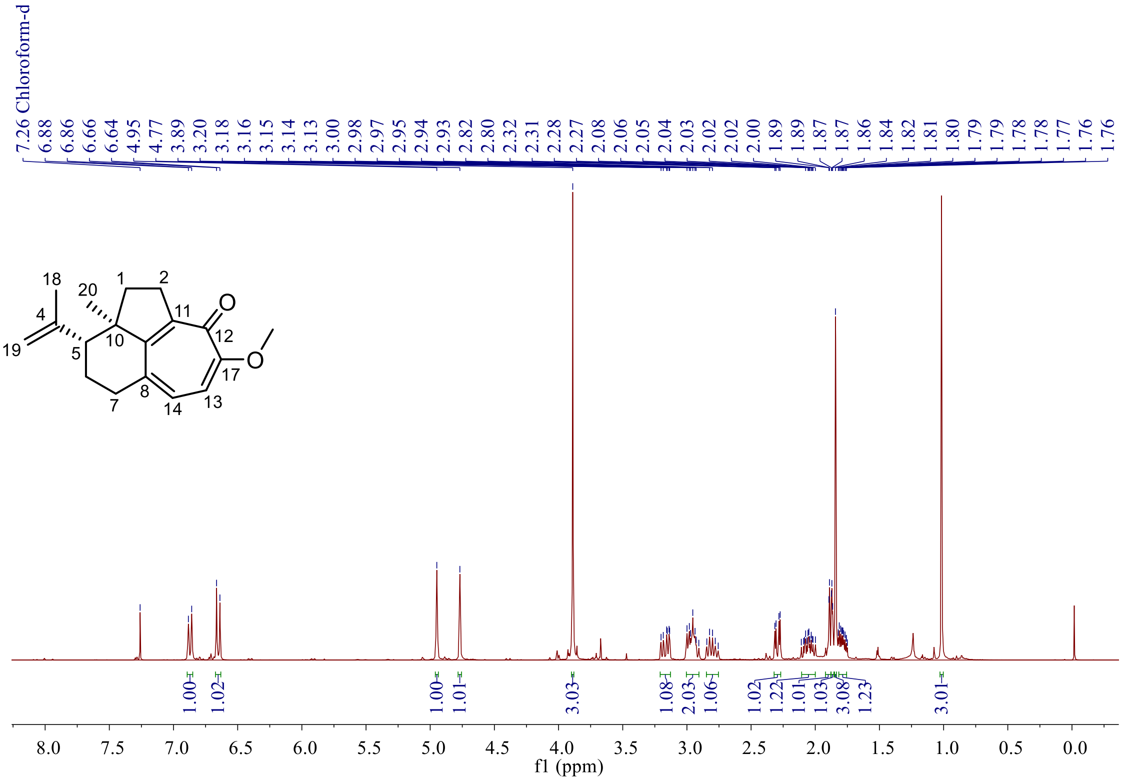

Figure S5. ^{13}C NMR and DEPT 135° spectra of **1** in $CDCl_3$ (100 MHz)

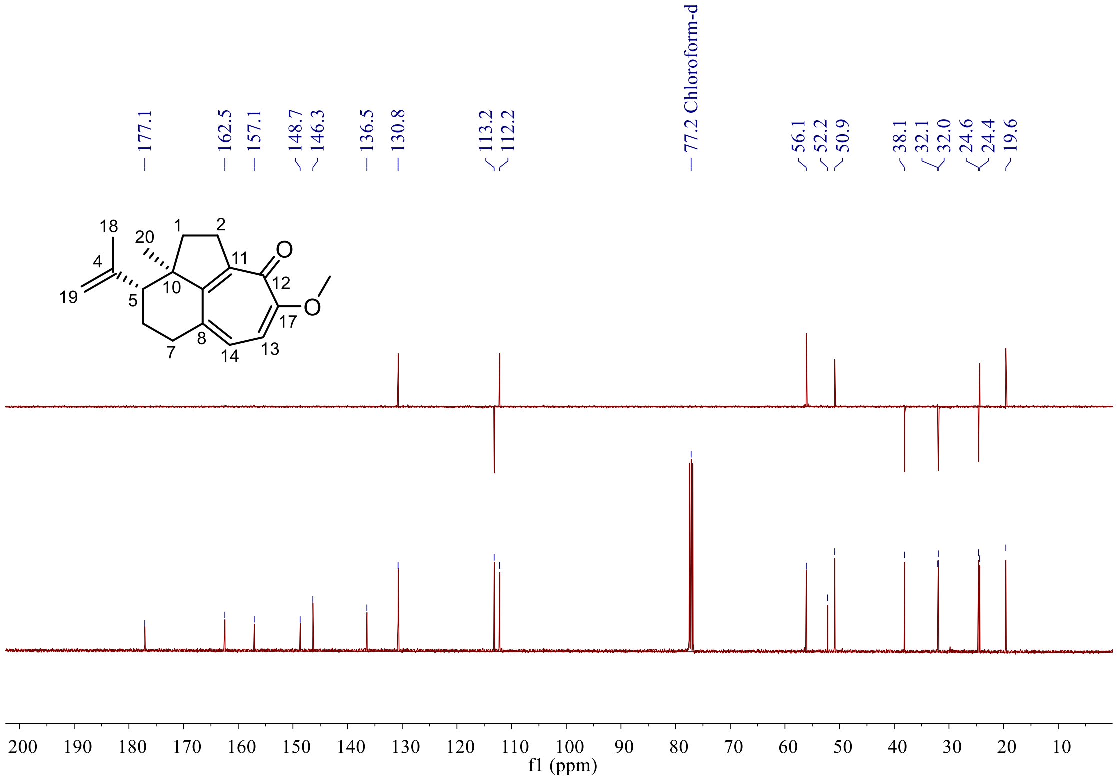

Figure S6. ^{1}H–^{1}H COSY spectrum of **1** in $CDCl_3$ (400 MHz)

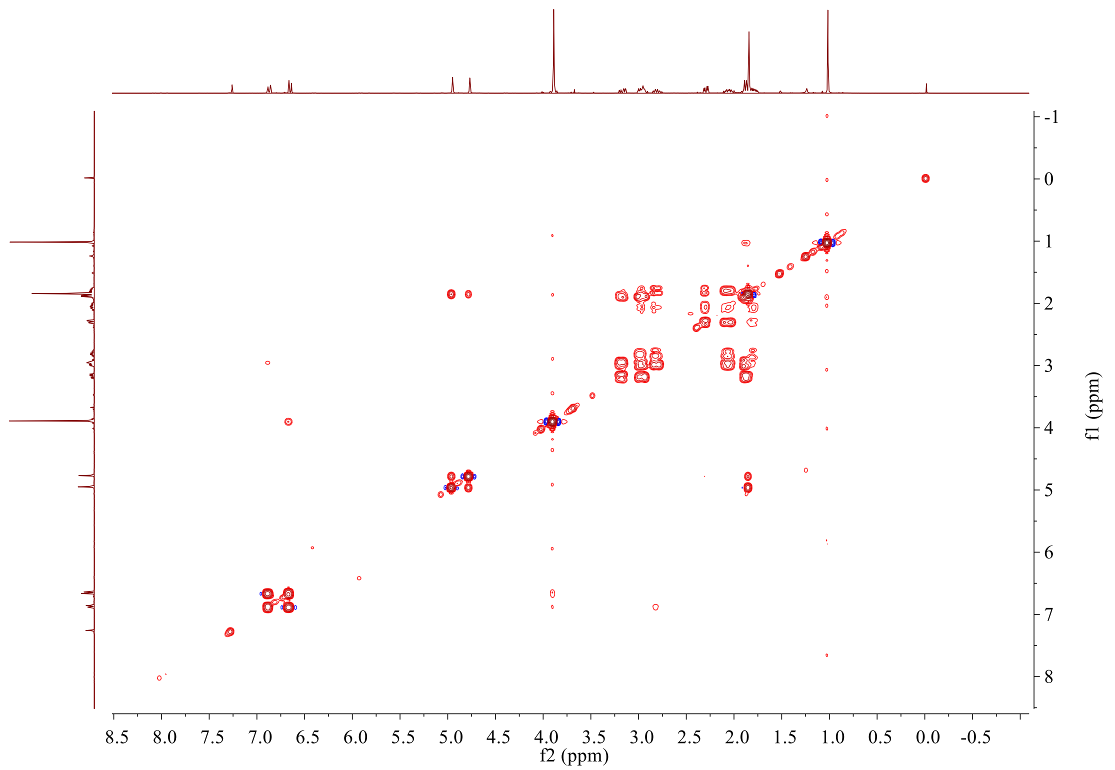

Figure S7. HSQC spectrum of **1** in $CDCl_3$ (400 MHz)

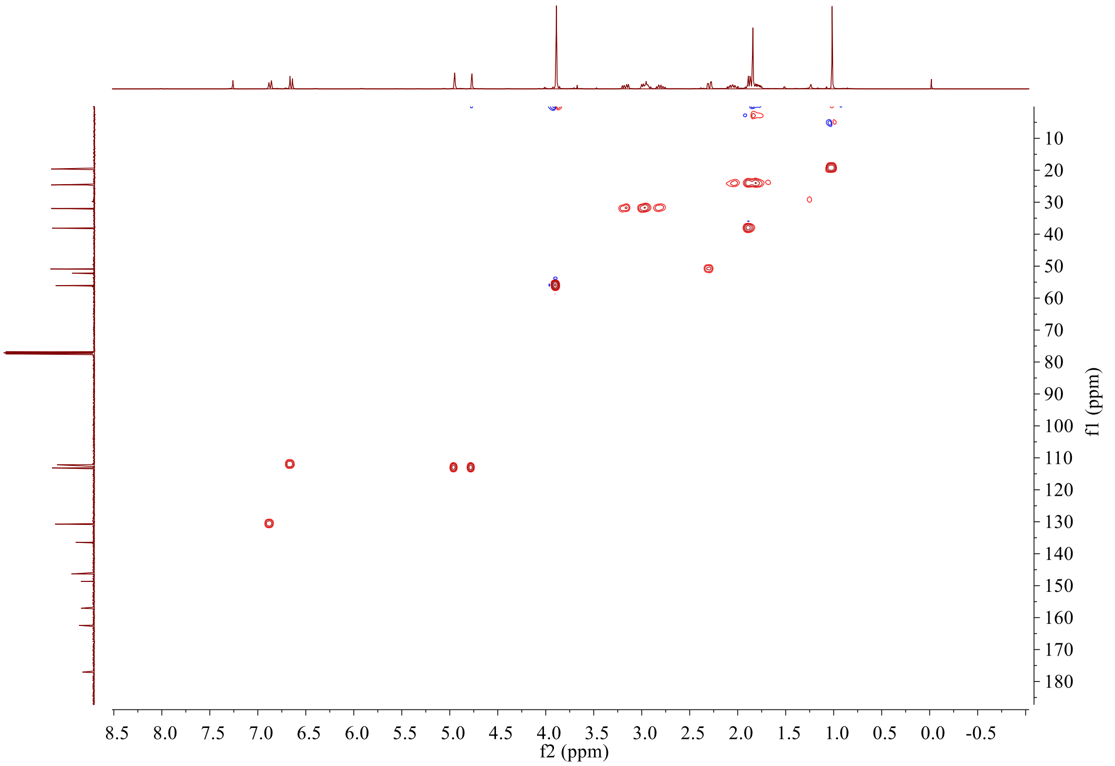

Figure S8. HMBC spectrum of **1** in $CDCl_3$ (400 MHz)

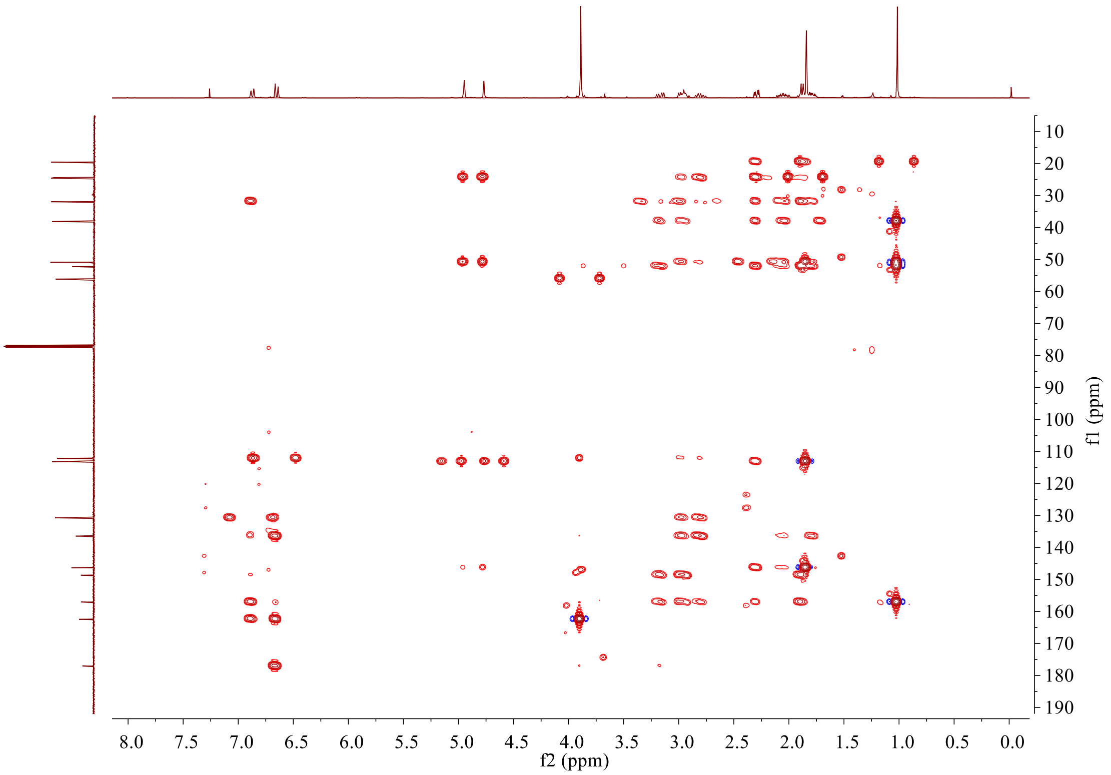

Figure S9. NOESY spectrum of **1** in $CDCl_3$ (400 MHz)

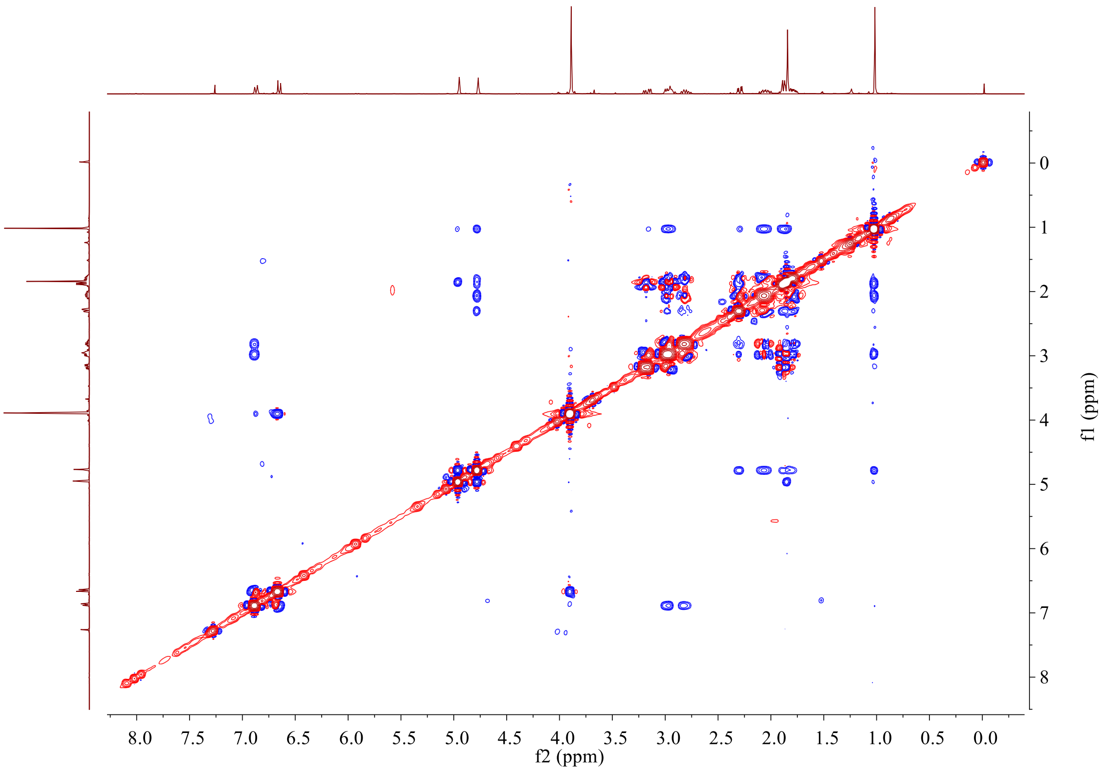

Figure S10. HRESIMS spectrum of **1**

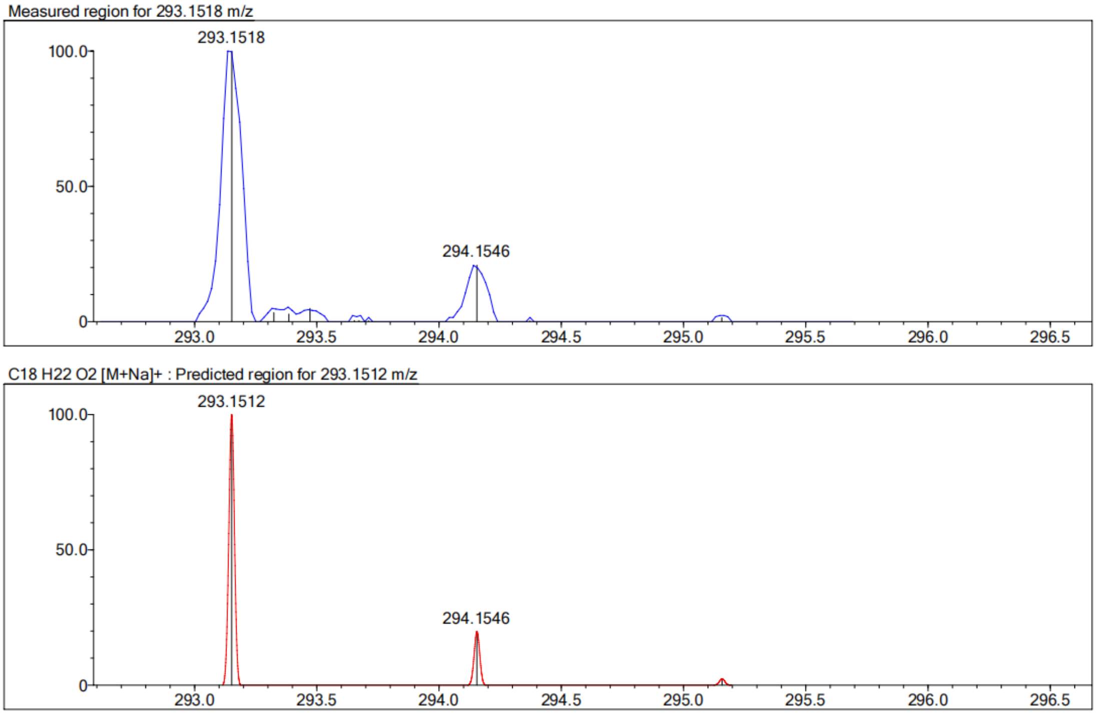

Rank	Score	Formula (M)	Ion	Meas. m/z	Pred. m/z	Df. (mDa)	Df. (ppm)	Iso	DBE
1	72.76	C18 H22 O2	[M+Na]+	293.1518	293.1512	0.6	2.05	74.73	8.0

Figure S11. IR spectrum of **1**

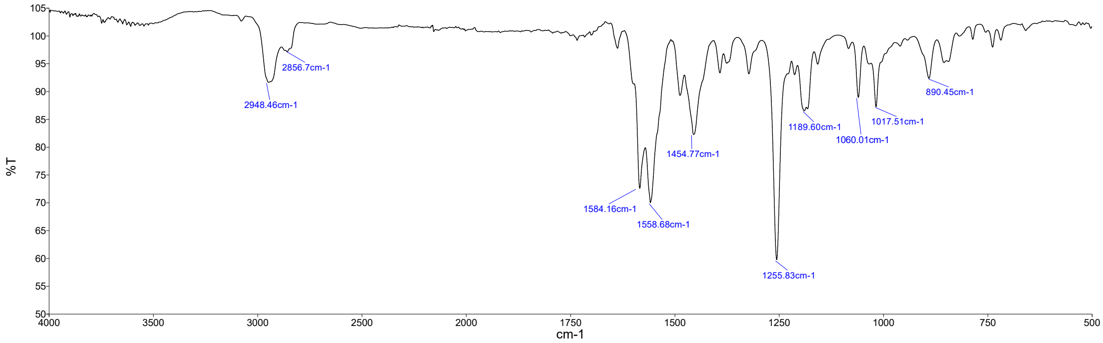

Figure S12. ^{1}H NMR spectrum of **2** in $CDCl_3$ (400 MHz)

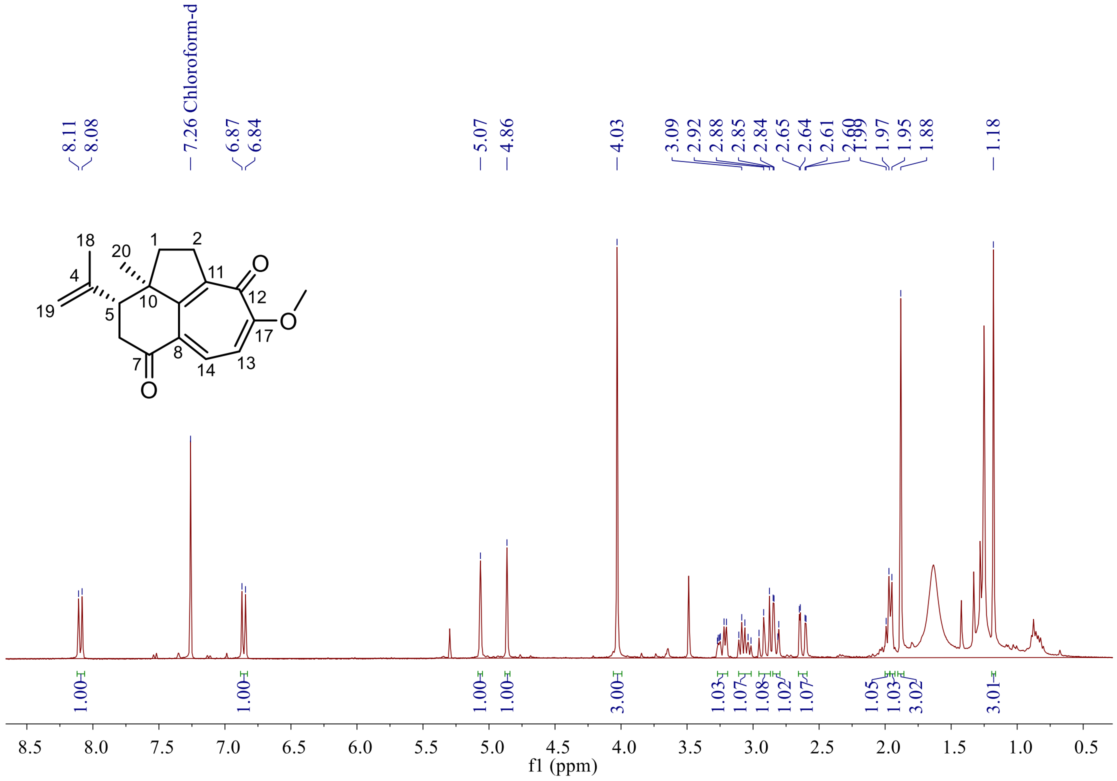

Figure S13. ^{13}C NMR and DEPT 135° spectra of **2** in $CDCl_3$ (100 MHz)

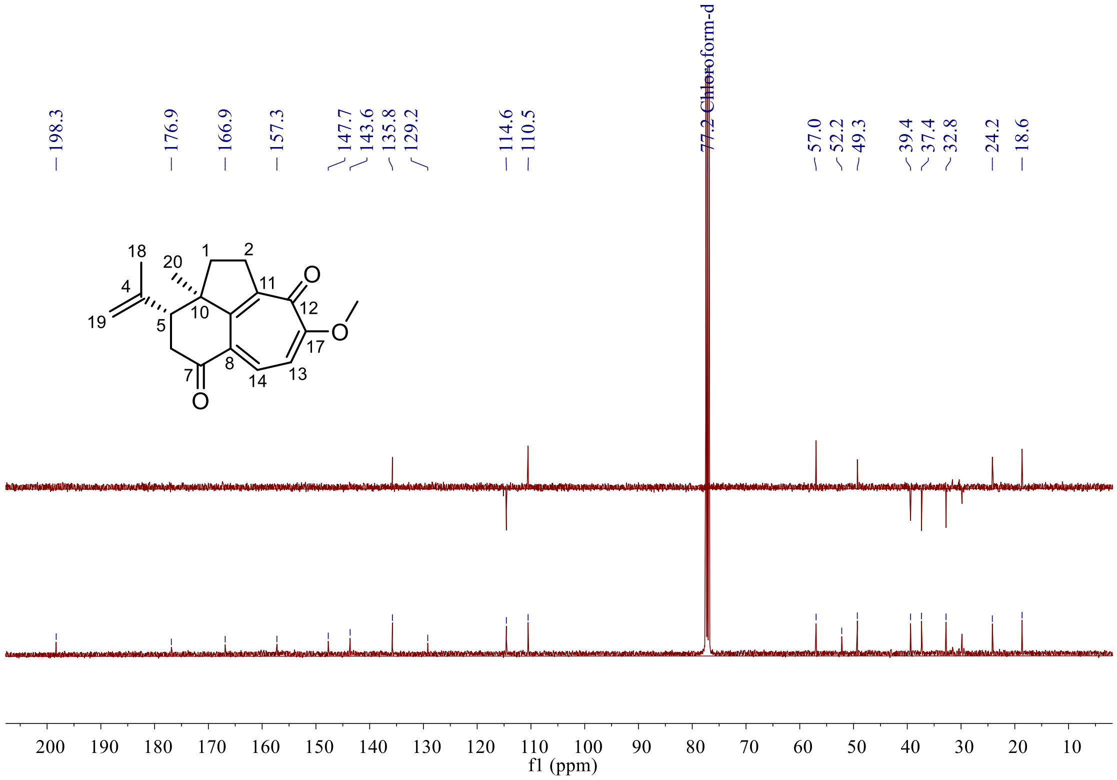

Figure S14. ^{1}H–^{1}H COSY spectrum of **2** in $CDCl_3$ (400 MHz)

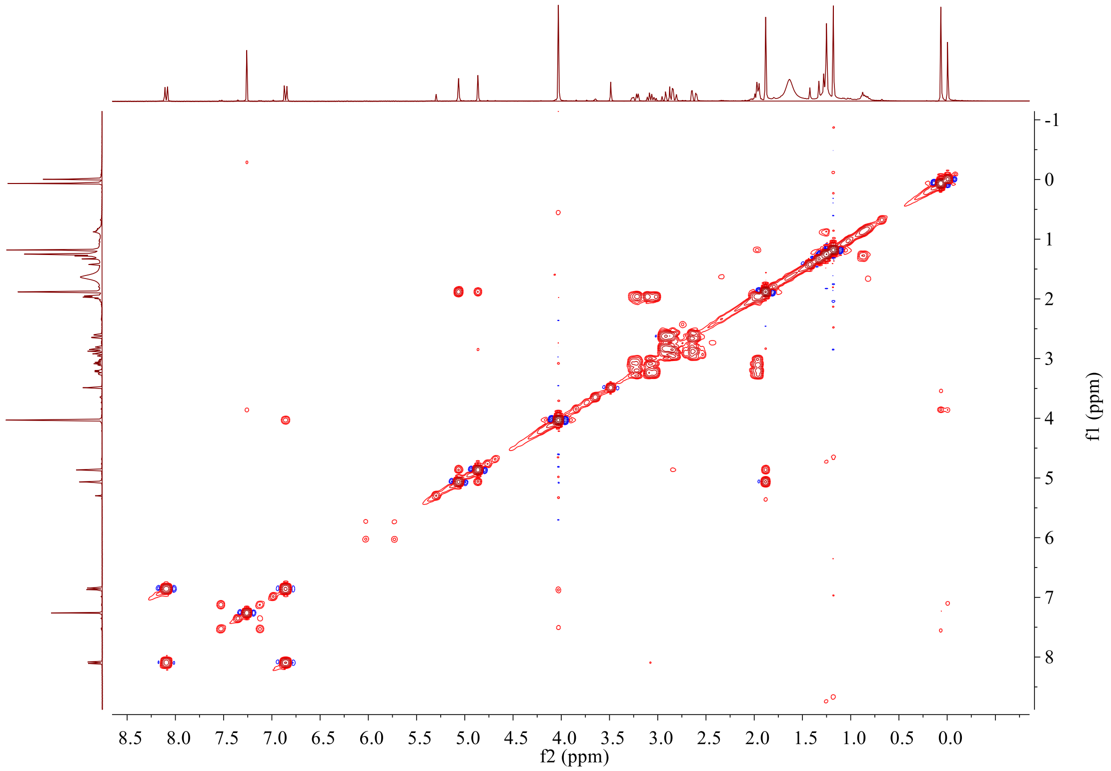

Figure S15. HSQC spectrum of **2** in $CDCl_3$ (400 MHz)

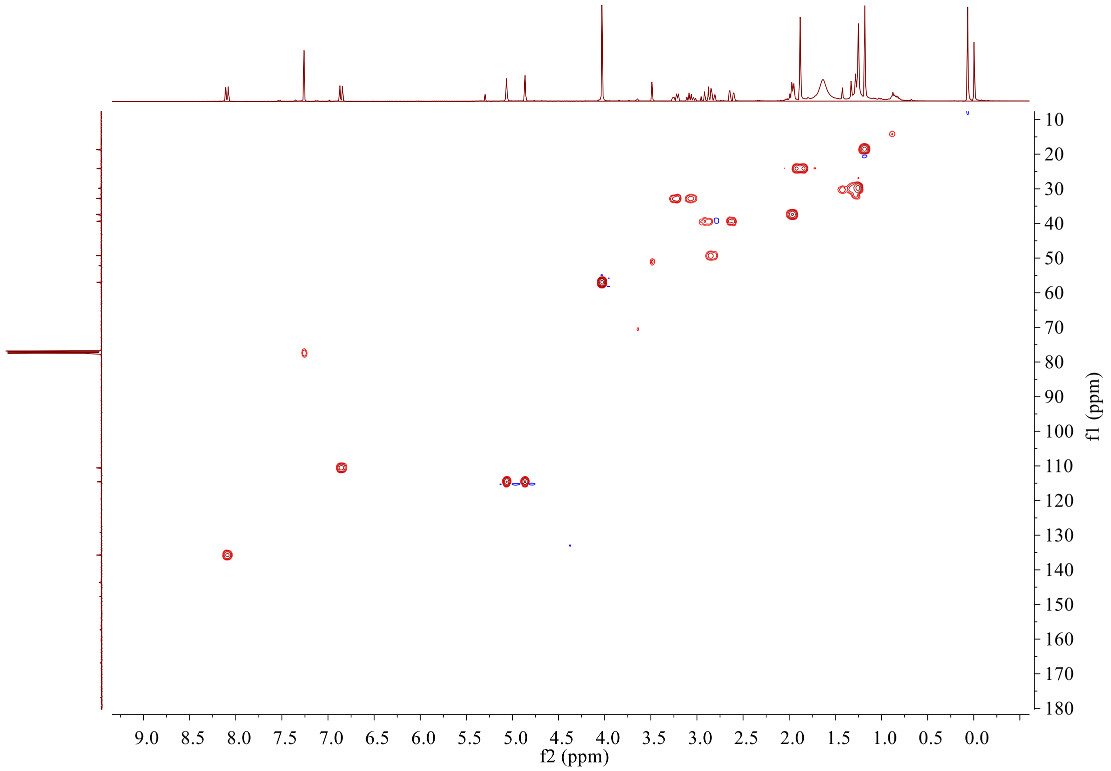

Figure S16. HMBC spectrum of **2** in $CDCl_3$ (400 MHz)

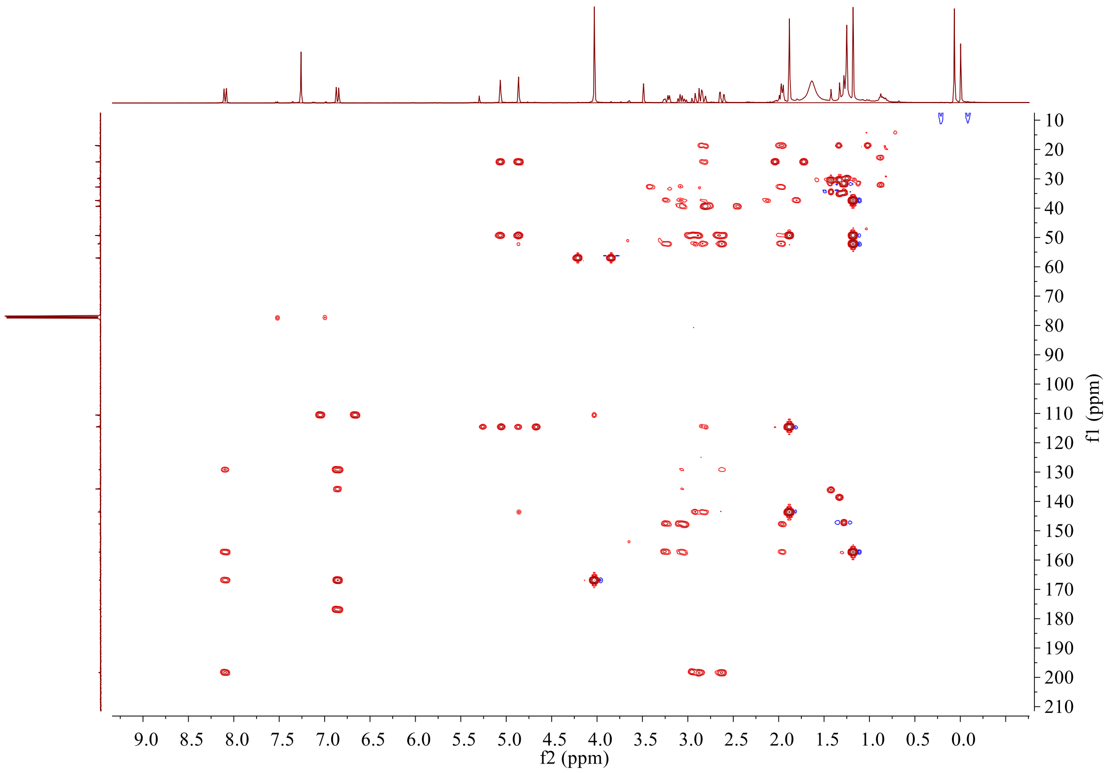

Figure S17. NOESY spectrum of **2** in $CDCl_3$ (400 MHz)

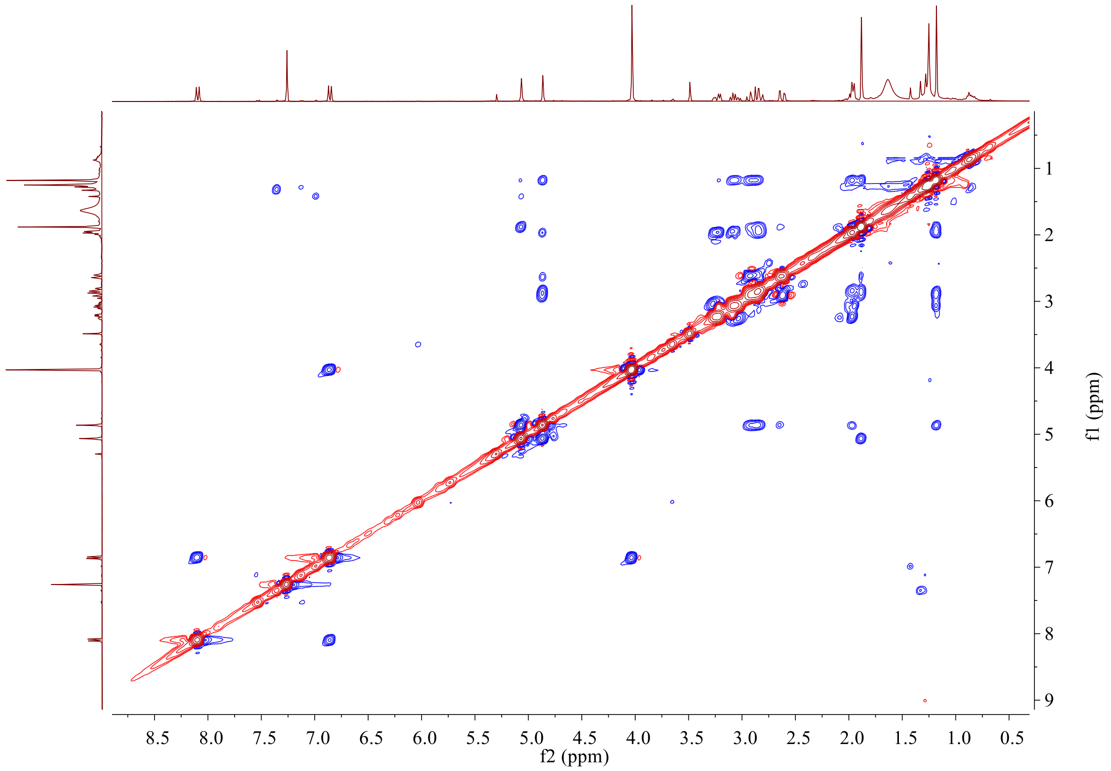

Figure S18. HRESIMS spectrum of **2**

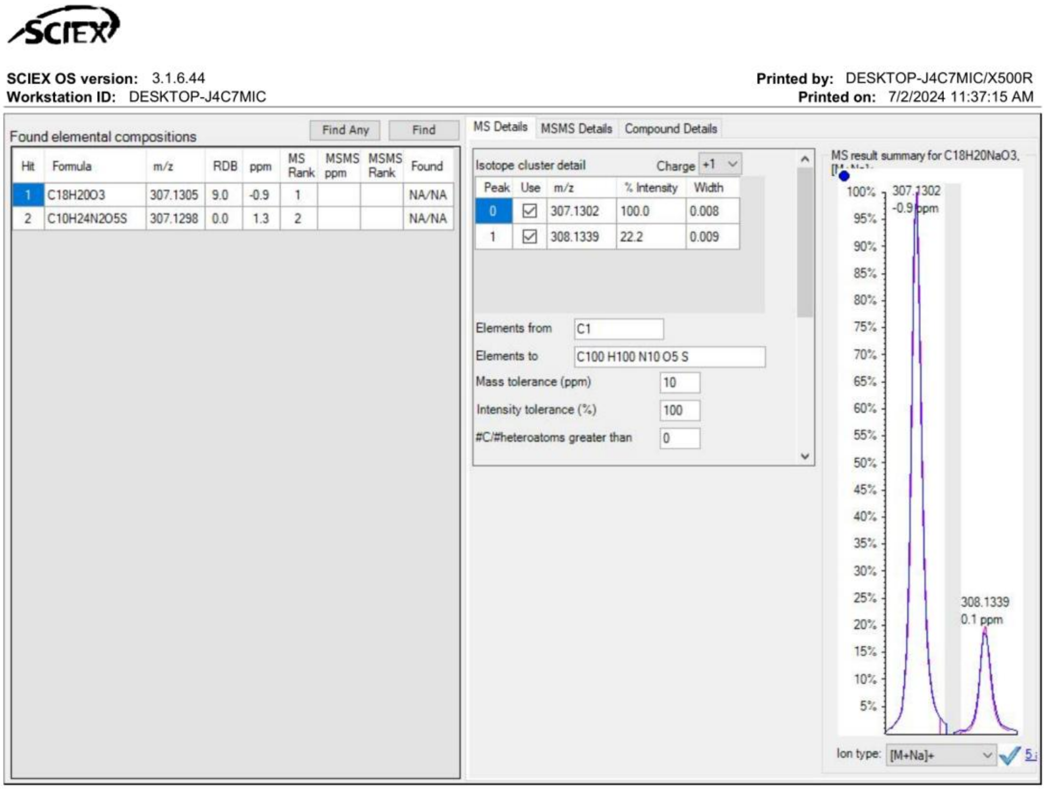

Figure S19. IR spectrum of **2**

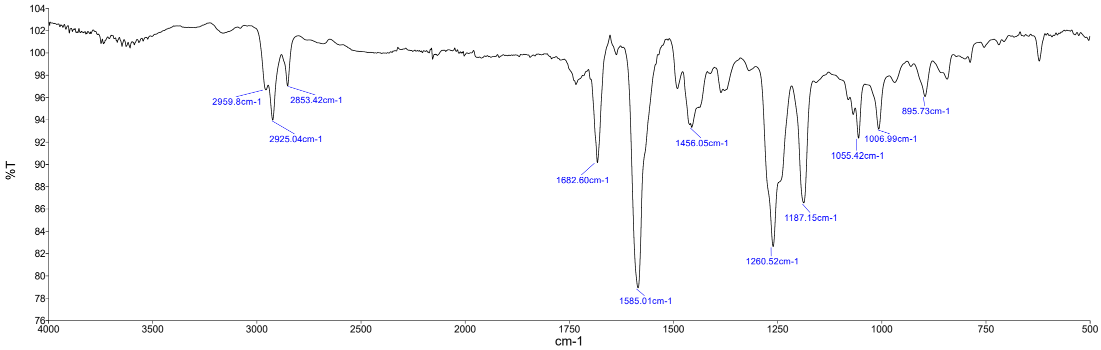

Figure S20. ^{1}H NMR spectrum of **3** in $CDCl_3$ (400 MHz)

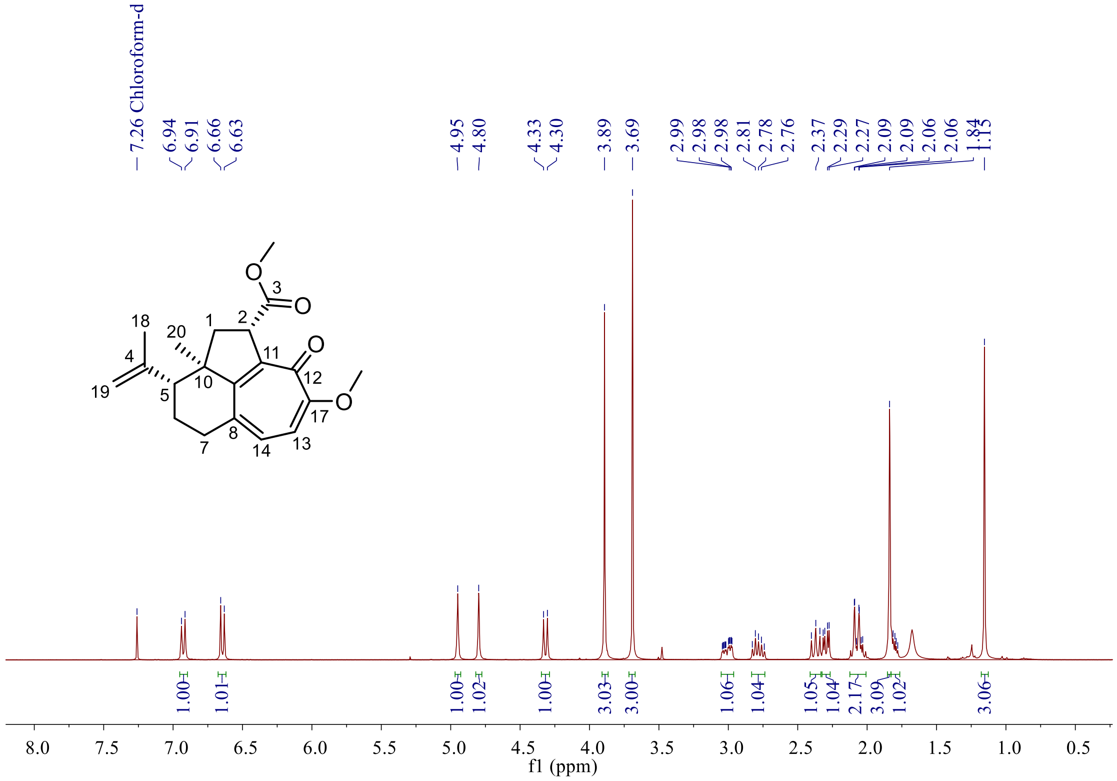

Figure S21. ^{13}C NMR and DEPT 135° spectra of **3** in $CDCl_3$ (100 MHz)

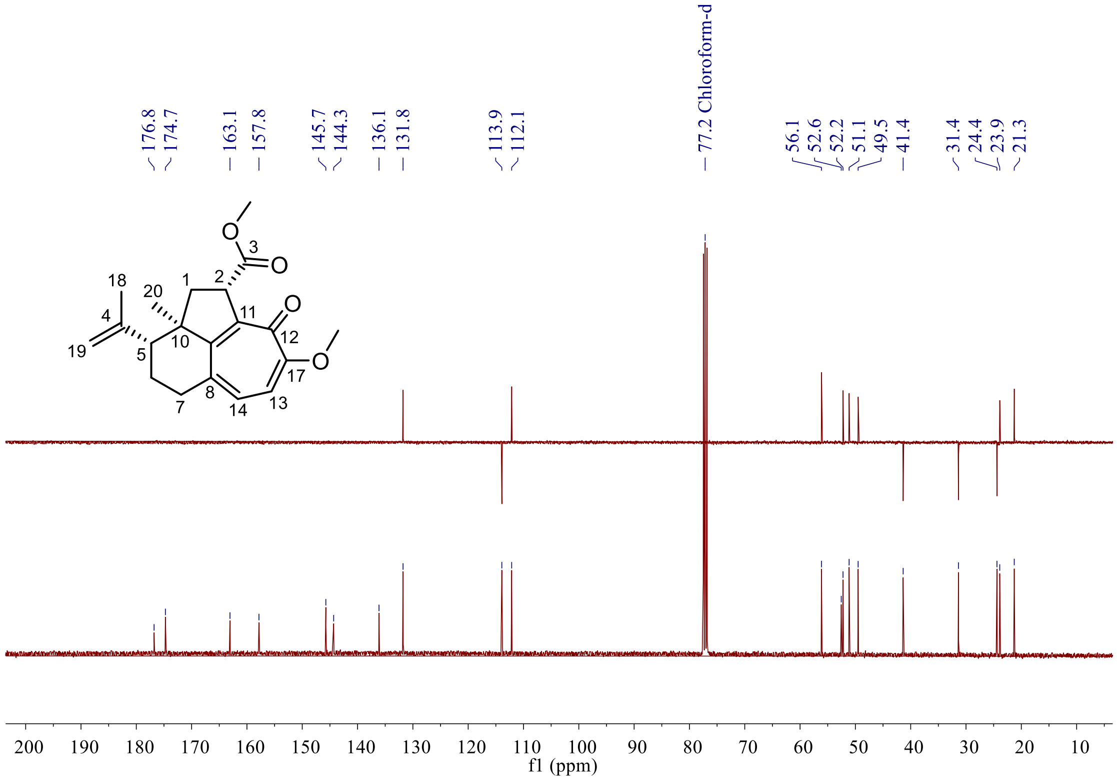

Figure S22. ^{1}H–^{1}H COSY spectrum of **3** in $CDCl_3$ (400 MHz)

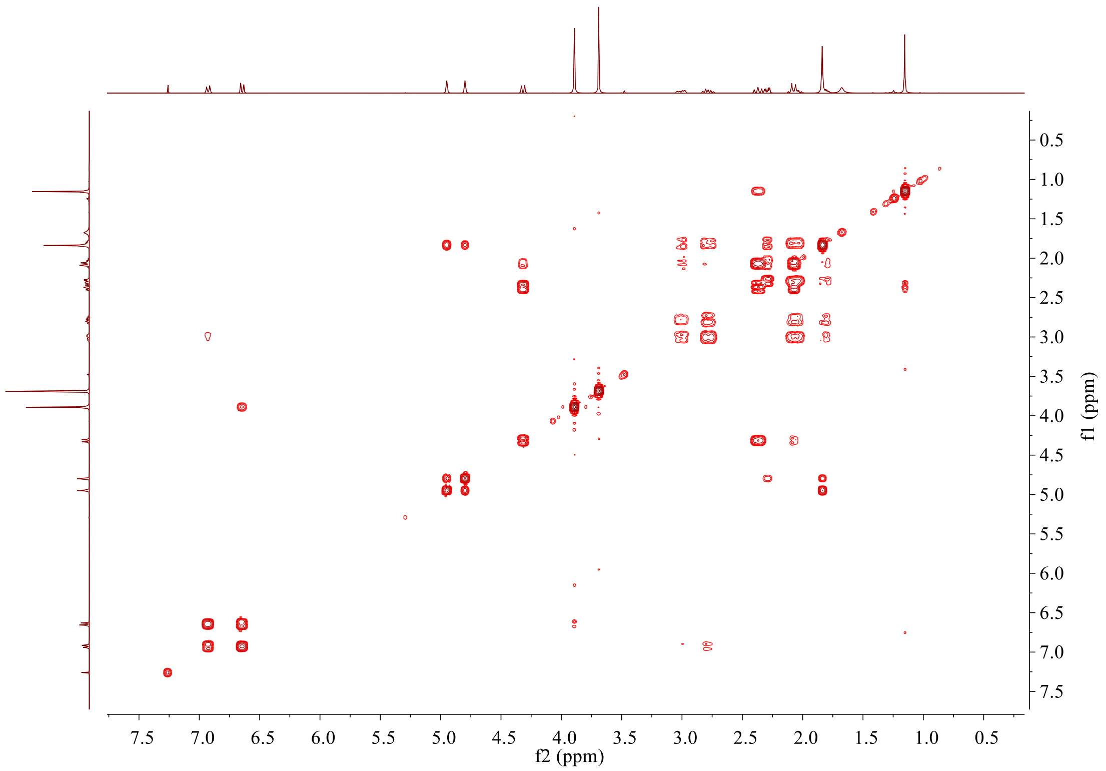

Figure S23. HSQC spectrum of **3** in $CDCl_3$ (400 MHz)

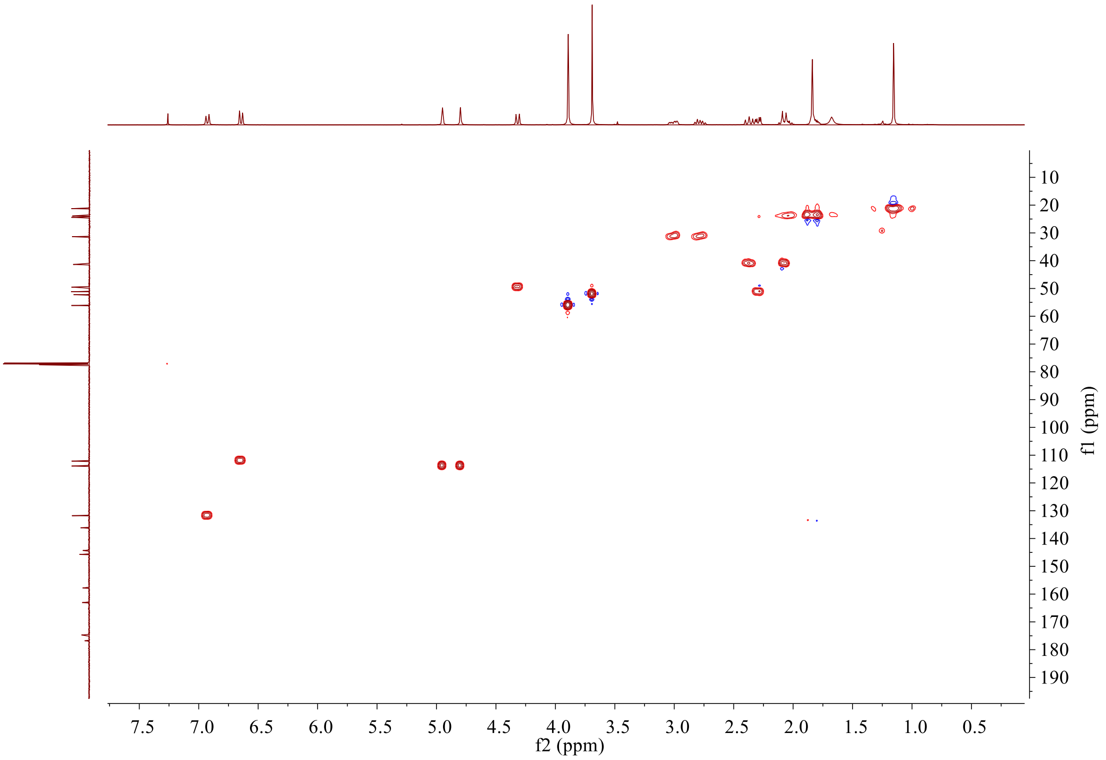

Figure S24. HMBC spectrum of **3** in $CDCl_3$ (400 MHz)

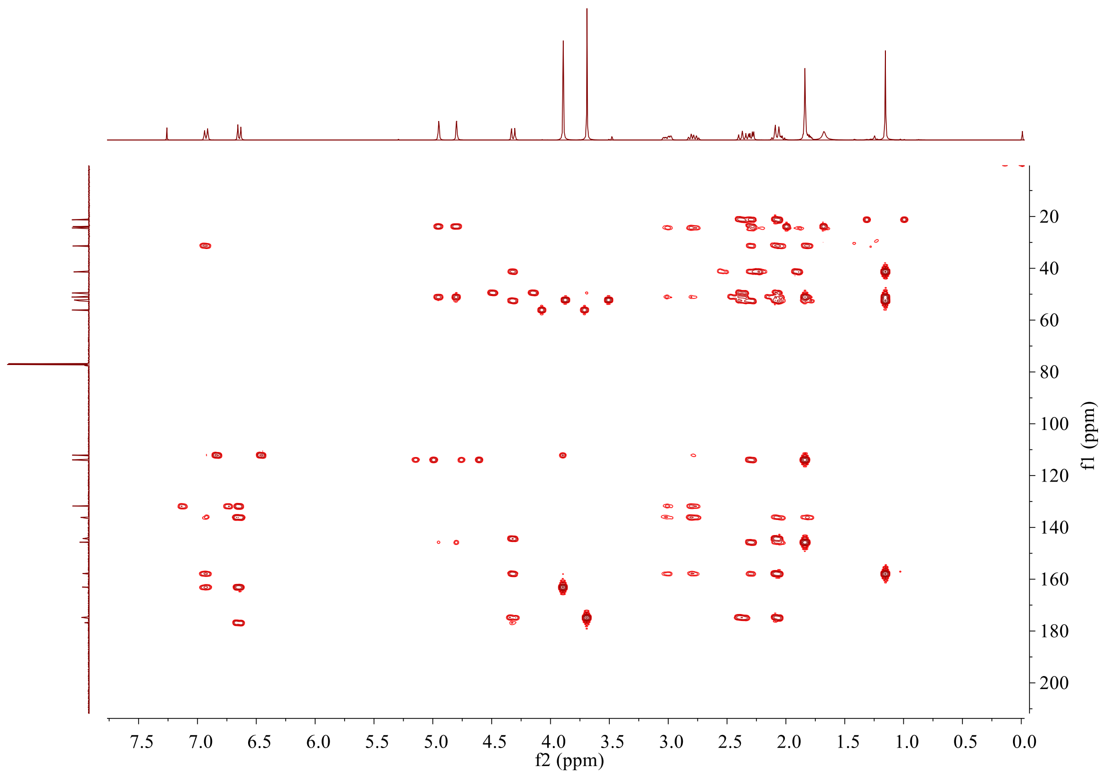

Figure S25. NOESY spectrum of **3** in $CDCl_3$ (400 MHz)

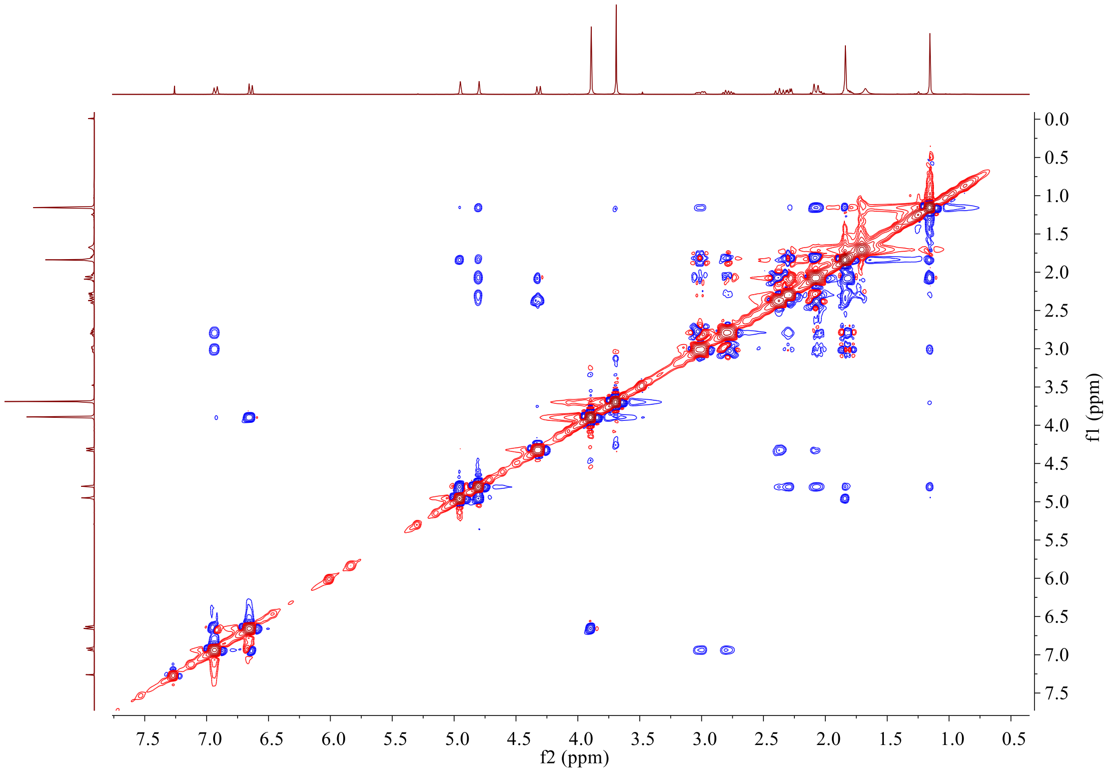

Figure S26. HRESIMS spectrum of **3**

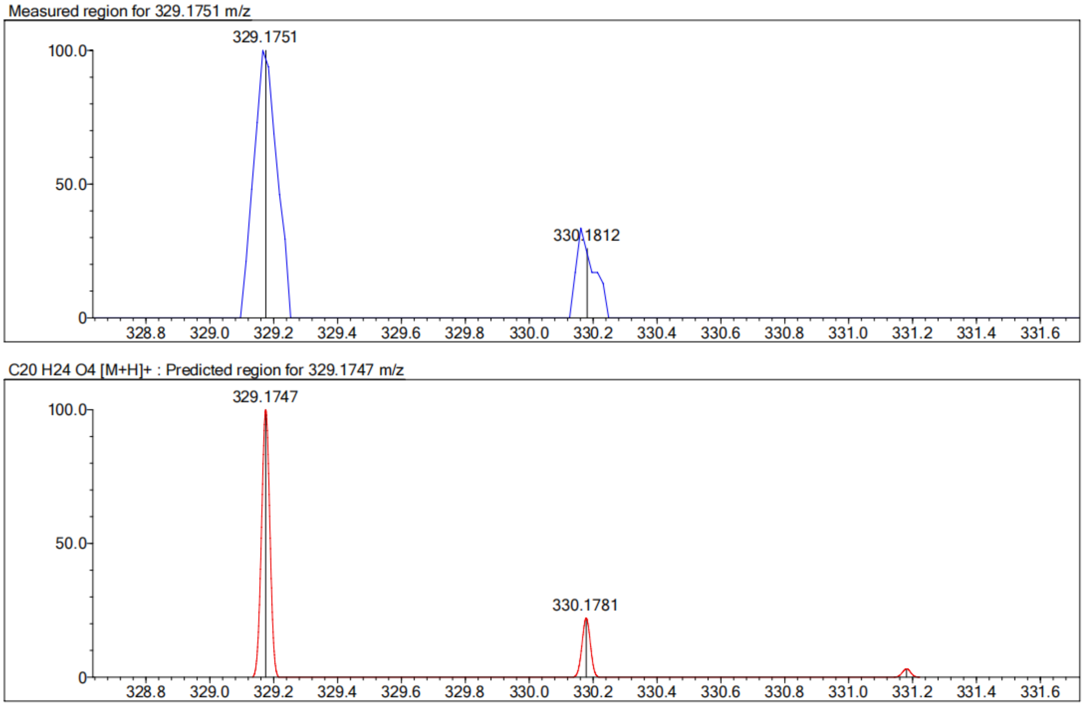

Rank	Score	Formula (M)	Ion	Meas. m/z	Pred. m/z	Df. (mDa)	Df. (ppm)	Iso	DBE
1	58.24	C20 H24 O4	[M+H]+	329.1751	329.1747	0.4	1.22	58.56	9.0

Figure S27. IR spectrum of **3**

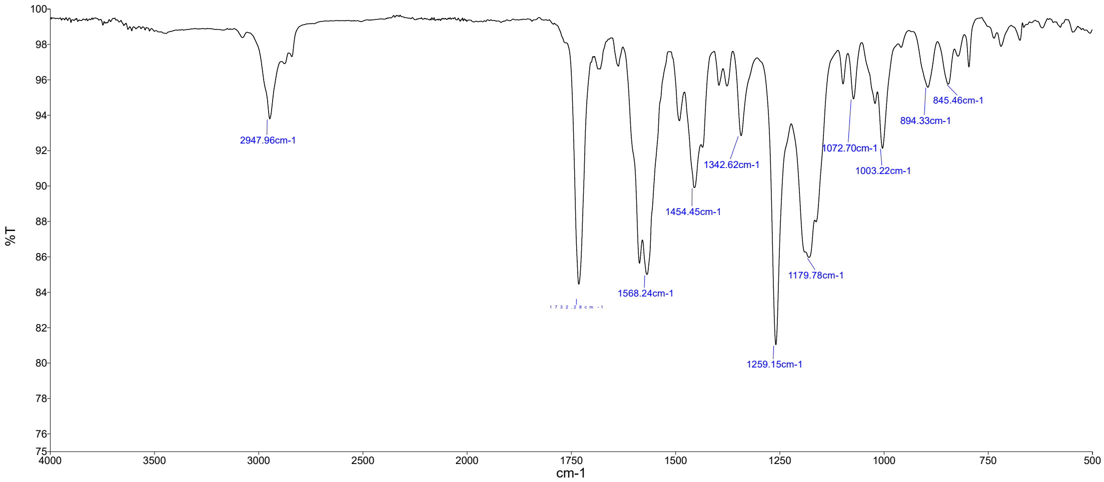

Supplement: Supplementary file 1 — Additional file 1. The 1D and 2D NMR, HRESIMS, and IR spectra of 1 − 3, and ECD calculations data for 2 and 3. [file 13659_2025_548_MOESM1_ESM.pdf]
